# Proteome Dynamics in iPSC-Derived Human Dopaminergic Neurons

**DOI:** 10.1016/j.mcpro.2024.100838

**Published:** 2024-09-07

**Authors:** Claudia Cavarischia-Rega, Karan Sharma, Julia C. Fitzgerald, Boris Macek

**Affiliations:** 1 Quantitative Proteomics, Department of Biology, Interfaculty Institute of Cell Biology, University of Tübingen, Tübingen, Germany; 2 Department of Neurodegeneration, Hertie Institute for Clinical Brain Research, University of Tübingen, Tübingen, Germany

**Keywords:** dopaminergic neurons, iPSC, proteomics, protein turnover, axon

## Abstract

Dopaminergic neurons participate in fundamental physiological processes and are the cell type primarily affected in Parkinson’s disease. Their analysis is challenging due to the intricate nature of their function, involvement in diverse neurological processes, and heterogeneity and localization in deep brain regions. Consequently, most of the research on the protein dynamics of dopaminergic neurons has been performed in animal cells *ex vivo*. Here we use iPSC-derived human mid-brain–specific dopaminergic neurons to study general features of their proteome biology and provide datasets for protein turnover and dynamics, including a human axonal translatome. We cover the proteome to a depth of 9409 proteins and use dynamic SILAC to measure the half-life of more than 4300 proteins. We report uniform turnover rates of conserved cytosolic protein complexes such as the proteasome and map the variable rates of turnover of the respiratory chain complexes in these cells. We use differential dynamic SILAC labeling in combination with microfluidic devices to analyze local protein synthesis and transport between axons and soma. We report 105 potentially novel axonal markers and detect translocation of 269 proteins between axons and the soma in the time frame of our analysis (120 h). Importantly, we provide evidence for local synthesis of 154 proteins in the axon and their retrograde transport to the soma, among them several proteins involved in RNA editing such as ADAR1 and the RNA helicase DHX30, involved in the assembly of mitochondrial ribosomes. Our study provides a workflow and resource for the future applications of quantitative proteomics in iPSC-derived human neurons.

## Highlights

• Deep proteome analysis of human iPSC-derived dopaminergic neurons.
• Determination of half-life of more than 4300 proteins by dynamic SILAC.
• Measurement of local protein synthesis and trafficking between axons and soma.
• Resource for future applications of quantitative proteomics in neurons.

## Graphical Abstract

**Figure:**
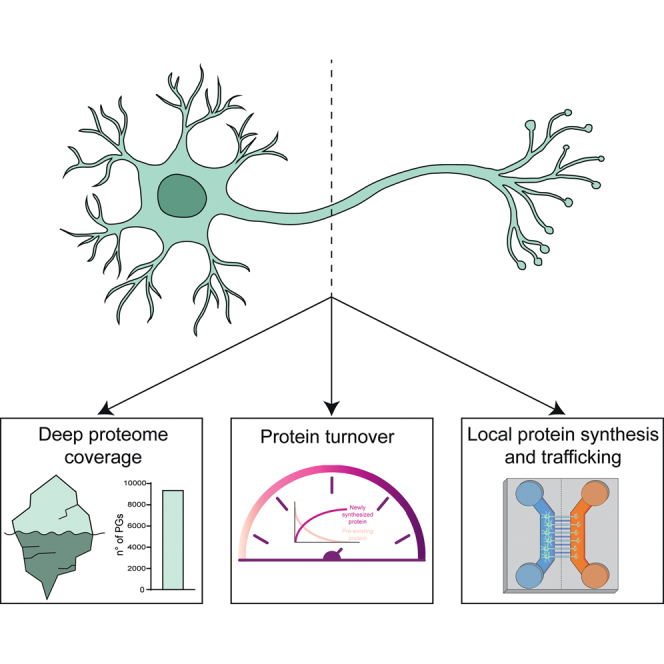

## In Brief

Our study provides in-depth proteome analysis of iPSC-derived human dopaminergic neurons (hDaNs). We use dynamic SILAC to measure half-lives of ∼4300 proteins. We apply differential SILAC labeling in microfluidic chambers to reveal axon-specific proteome and provide evidence for local protein synthesis and trafficking between axons and the soma. This dataset serves as a reference for studying axonal proteins and a resource for researchers working on protein biology of hDaNs.

Dopaminergic neurons are a subset of neurons within the central nervous system characterized by their synthesis and release of dopamine, a pivotal neurotransmitter (1). They are predominantly localized in discrete brain regions, notably the *substantia nigra* (SN) and the ventral tegmental area (2), and they participate in fundamental physiological processes that include motor control, reward mechanisms, motivation, and mood regulation (3). The study of dopaminergic neurons poses significant challenges owing to their heterogeneity and localization in deep brain structures, impeding direct access for experimental interventions (2, 4). Dopaminergic neurons are especially relevant in the context of Parkinson’s disease (PD), which is characterized by their selective loss in the SN region of the midbrain (5). Recently, extensive investigations into the proteome of this specific neuronal subtype in PD was performed using *post mortem* brain tissue from patients (6), but there are no dynamic studies of cultured human dopaminergic neurons, which are a mainstay model system.

Quantitative proteomics has long since contributed to our understanding of proteomic alterations associated with neurodegenerative disorders, including PD, Huntington’s disease, Schizophrenia, and Alzheimer’s disease (AD) (6, 7, 8, 9, 10). Several proteomics studies have scrutinized the bulk proteome and post-translational modification (PTMome) (11) of human induced pluripotent stem cell (iPSC)-derived mid-brain–specific dopaminergic neurons (hDaNs) models of familial (12, 13, 14, 15) and sporadic PD (16). Other studies have focused on the synaptic proteome of striatal dopaminergic terminals in mice (17) and, recently, the distinct protein expression profiles in the axons and soma of dopaminergic neurons in mice (18).

Protein turnover is defined by the rates of protein synthesis and degradation, and it is previously shown to be dependent on the cell type (19). This underscores a necessity for a reference dataset that focuses on the turnover of the proteins in hDaNs. Dynamic Stable Isotope Labeling with Amino acids in Cell culture (SILAC) has emerged as a powerful tool for investigating protein turnover (20, 21, 22). Studies on protein turnover in mice (23, 24) and rats (25, 26, 27) have provided crucial insights into the cellular dynamics of dopaminergic neurons, yet further work in human cells is needed to bring forward methods and resources for translational research and preclinical studies.

Axons are elongated subcellular compartments protruding from the cell body (soma). Proper arborization of axons and synapses are essential for neuronal function. Increasing evidence suggests that early primary cilia impairments (affecting neuronal development, axon pathfinding, and migration) contribute to PD etiology (28, 29, 30). Axons possess the machinery for protein synthesis, including mRNA, ribosomes, and other necessary factors (31, 32, 33, 34). Regulation of the local translation of mRNAs and protein degradation enables spatial and temporal control of the axonal proteome in response to multiple stimuli (35). Local translation in axons is especially important for axonal guidance and synaptic plasticity, due to the considerable distance from the soma, the primary site for protein synthesis in neurons (36). Recent work has also highlighted the importance of local translation in axons to support mitophagy (37), a form of mitochondrial quality control associated with aging and PD (38, 39, 40). The study of local protein synthesis using pulsed SILAC in primary neurons derived from *Xenopus* embryos has shown that different guidance cues can induce rapid synthesis of new proteins in the axons within minutes (41). Microfluidic devices have been used to study axonal injury and regeneration, where the authors also demonstrated their utility in providing fluidic isolation between the wells (42). These devices have also been used for targeted studies, for example, to investigate the effect of a mutation in the neurofilament light gene in human iPSC-derived motor neurons (43) or to study the replication of mitochondrial DNA in the axons, operating independently of the soma in primary neurons from mice (44). Furthermore, there is a large body of work demonstrating retrograde transport of murine proteins (Importin β1, Stat3, mTOR, CREB, and ATF4) synthesized in axons in response to injury stimuli (45, 46, 47, 48, 49).

In this study, we employ a workflow based on mass spectrometry and stable isotope labeling of neurons, in combination with cell culture in microfluidic devices, to uncover protein composition, turnover dynamics, and spatial regulation of proteins in untreated human iPSC-derived hDaNs from a healthy donor. We cover approximately 9400 proteins and use dynamic SILAC to measure the half-life of more than 4300 proteins. We employ differential dynamic SILAC labeling in combination with microfluidic devices to analyze local protein synthesis and transport between axons and soma. We report novel axonal markers and detect translocation of proteins between axons and the soma. Importantly, we provide evidence for local synthesis of over 150 proteins in the axon and their retrograde transport to the soma.

## Experimental Procedures

### Experimental Design and Statistical Rationale

All experiments were performed in biological triplicates, all of which presented independent differentiations of iPSC-derived hDaNs. For the turnover dataset, only proteins with detectable label incorporation in at least four out of five times points in at least two out of three replicates were taken into consideration. For the data from microfluidic devices, only proteins with label incorporation in two out of three replicates were taken into consideration. These filtering criteria were chosen as a compromise between statistical stringency and number of data points that remain after filtering. Statistical analysis was performed with two-tailed unpaired *t* test in GraphPad Prism. Significance is indicated by asterisks: ∗*p* ≤ 0.05, ∗∗*p* ≤ 0.01, ∗∗∗*p* < 0.001. For the pathway enrichment analysis, a Fisher exact test with a false discovery rate (FDR) < 0.05 was performed.

### Generation of neural progenitor cells and Differentiation to hDaNs

#### Human Dopaminergic Neuron Differentiation

An iPSC line derived from a healthy female donor was previously generated and characterized by Schwarz, et.al., 2021 (50). Briefly, iPSCs were used to generate human dopaminergic neurons (hDaNs) *via* neural progenitor cell intermediates using chemical induction as described in Schwarz, *et. al*., 2022 (51) and based on a protocol described by Reinhardt, *et. al*., 2013 (52) with slight modification (13). Experiments were performed at day 18 (mature hDaNs) and the cells were harvested at day 23 for proteomic analysis.

#### Characterization of hDaNs

The efficiency of differentiation was validated with immunofluorescence data (Supplemental Fig. S5, *A* and *B*) and analysis of the proteomic dataset for dopaminergic markers compared with markers of mature neurons and their subtypes (TH for dopaminergic neurons, TPH for serotonergic neurons, GAD1 for GABAergic neurons, and ChAT for cholinergic neurons) (Supplemental Fig. S5*C*). These data are in line with previous, published characterization of mid-brain–specific dopaminergic neurons differentiated *via* neuronal progenitors using the same protocol and same cells (13, 52).

#### Culturing hDaNs in Microfluidic Devices

On day 9 post-differentiation start, cells were dissociated using Accumax, Cell Dissociation Solution (#P10-21200, PAN-Biotech) and plated onto XonaChips XC900 (# XC900, Xona Microfluidics) at a density of 70,000 cells per chamber in the proximal or somatodendritic well (140,000 cells in total), according to the manufacturer’s protocol. hDaN differentiation was then induced and specific experiments were performed after maturation at day 18 post-differentiation start and harvested at the day 23 post-differentiation.

### Imaging

Eighteen days prior to imaging, 140,000 differentiating hDaNs (day 9 post differentiation start) were seeded on Matrigel-coated Xona XC900 Microfluidic chip. For labeling of mitochondria and neutral lipids, hDaNs (day 27 post differentiation start) were stained continuously at 37 °C with 5% CO2 with 100 nM MitoTracker deep red (#M22426, Thermo Fisher Scientific) and 4.77 μM Bodipy (#790389-500 MG, Sigma), respectively. For imaging, the medium was not replaced and a Leica DMi8 Microscope (4 × and 10× objective) with the LASX software was used.

For imaging on microscopy slides, ∼30,000 differentiating hDaNs (day 15 post differentiation start) were seeded on matrigel-coated coverslips. On day 18, the cells were fixed using 4% paraformaldehyde solution for 15 min at room temperature. Post fixation, the cells were permeabilized and blocked using a 1% bovine serum albumin (BSA) + 0.3% Triton X-100 solution in PBS for 1 h. The primary antibodies STMN2 (mouse, #sc-135620, SantaCruz), DHX30 (rabbit, ab254660, Abcam), and GAP43 (rabbit, #NB300-143SS, Novus) were added at 1:500 dilution in 1% BSA + 0.1% Triton X-100 solution in PBS overnight at room temperature. The following day, secondary antibodies goat anti-mouse 488 and goat anti-rabbit 488 were added at 1:500 in 1% BSA + 0.1% Triton X-100 solution in PBS for 1 h at room temperature protected from direct light. The cells were washed with PBS three times and incubated with Hoechst stain (#H3569, Molecular Devices) at 1:5000 in PBS for 5 min. The cells were then washed with PBS and mounted with Dako fluorescent mounting medium (#S3023, Agilent) and placed onto microscopy slides, sealed with nail polish, and stored at 4 °C prior to imaging. For imaging, Zeiss Imager.Z1 equipped with an ApoTome.2 and an AxioCam MRm was used and the images were captured using the 40× and 63× objective. ImageJ2 (Fiji) was used for processing the images.

For fixed cells imaging on microfluidic devices, the same fixation, permeabilization, blocking, and antibody addition protocol was followed as described above. Tyrosine hydroxylase antibody (mouse, #sc-25269, Santa Cruz) was used at 1:500 dilution. The secondary antibodies goat anti-mouse 488 was used at 1:500 dilution. Following antibodies staining, Hoechst stain was added followed by a PBS wash and Dako fluorescent mounting medium was added to the top wells as advised by Xona microfluidic manual for immunofluorescence staining. The images were captured using Leica DMi8 Microscope (4× and 10× objective). The images were processed using ImageJ2 (Fiji).

### Treatments

Dulbecco’s modified Eagle’s medium (DMEM)-SILAC (Sigma-Aldrich) lacking arginine and lysine supplemented with penicillin/streptomycin (100 U/ml, PAN) and stable isotope-encoded arginine and lysine was added to the cells. The ‘light’ SILAC media was supplemented with L-[^12^C_6_,^14^N_2_] lysine (Lys0) and L-[^12^C_6_,^14^N_4_] arginine (Arg0) (Cambridge Isotope Laboratories), whereas L-[^2^H_4_] lysine (Lys4) and L-[^13^C_6_] arginine (Arg6) were added to the ‘medium-heavy’ SILAC media and L-[^13^C_6_,^15^N_2_] lysine (Lys8) and L-[^13^C_6_,^15^N_4_] arginine (Arg10) to ‘heavy’ SILAC media.

For the turnover dataset, hDaNs (on day 18) were cultured in DMEM-SILAC heavy/medium-heavy media for 0, 6, 12, 48, 72, and 120 h and were all harvested on day 23. For the 120 h timepoint, hDaNs were cultured in SILAC medium on day 18, while for the 72 h timepoint, hDaNs were cultured in SILAC media on day 20 and similar for the other timepoints. This was done so that the cells were harvested on day 23, all at the same time and to avoid differences that could arise due to the cell’s maturation state.

For the experiments in microfluidic devices, after axons protruded through the microgroove barriers, DMEM-SILAC “medium-heavy” medium was added in the somatodendritic enriched well and DMEM-SILAC “heavy” was added in the axonal well for 12, 48, 72, and 120 h. At the somatodendritic (proximal) well, 150 μl media was added, while at the axonal (distal) well, 120 μl media was added to create hydrostatic pressure and ensure fluidic isolation between the two wells. The cells were harvested on day 23 for all timepoints.

To prevent protein translation, 100 μM cycloheximide (CHX) solution (#239765, Calbiochem) was used (33) and added to the somatodendritic well and/or to the axonal side.

### Sample Preparation for Mass Spectrometry Analysis

#### Cell Lysis

For data-independent acquisition (DIA) and turnover dataset, cells pellets were lysed with urea lysis buffer (6 M urea, 2 M thiourea, 60 mM Tris pH 8.0) and kept on ice for 20 min. DNA and RNA were removed from the cell lysate using benzonase (1 U/ml, Merck Millipore) for 10 min at room temperature (RT) and samples centrifuged for 10 min at 13,000 rpm and 4 °C.

Protein quantification was performed with Bradford reagent, using a standard curve with known concentrations of BSA, and the absorbance was measured at 595 nm.

#### Extraction from Microfluidic Devices

The cells were washed with PBS (#D8537, Sigma-Aldrich) followed by the addition of 30 μl of urea lysis buffer (6 M urea, 2 M thiourea, 60 mM Tris pH 8.0) into each well. A 10 μl pipette tip was used to scrape the surface of the device and the lysates were collected and stored at −80 °C until further analysis.

#### Protein In-Solution Digestion

Proteins were reduced with 10 mM of DTT for 1 hour, alkylated with 55 mM iodoacetamide for 1 hour, and digested with Lys-C (Lysyl Endopeptidase, Wako Chemicals) for 3 hours at RT. Afterwards, four volumes of 10 mM ammonium bicarbonate were added and proteins were digested with trypsin (Promega Corporation) overnight. To stop the digestion, 1% TFA was added.

#### High pH Reverse-Phase Chromatography

For the DIA and turnover datasets, samples were fractionated with the Pierce High pH Reversed-Phase Peptide Fractionation Kit (Thermo Fisher Scientific, Kit #84868). First, the samples were purified by solid phase extraction on Sep-Pak C18 cartridges (Waters). Hundred micrograms of peptides were loaded in the conditioned spin columns and separated into nine fractions according to the hydrophobicity. Elution was done with a steep gradient of acetonitrile and ammonia (5%-50% acetonitrile in 10 mM NH_4_OH). Finally, fractions were acidified to pH to <2.7 with TFA, dried by vacuum centrifugation, and purified on C18 StageTips prior LC-MS/MS measurements.

### LC–MS Measurement

Peptide samples were measured on an Exploris 480 mass spectrometer (Thermo Fisher Scientific) online-coupled to an Easy-nLC 1200 UHPLC (Thermo Fisher Scientific). Peptides were separated using a 20-cm-long, 75-μm-inner diameter analytical HPLC column (ID PicoTip fused silica emitter; New Objective) packed in-house with ReproSil-Pur C18-AQ 1.9-μm silica beads (Dr Maisch GmbH).

For DIA analysis, samples were measured using 90 min LC gradient which was optimized for each HpH fraction. The full scan range was set to 400 to 1000 m/z at a resolution of 120k. Fragment ions were analyzed in 40 DIA windows, with an isolation window of 15 Th (1 Th overlap), at a resolution of 30,000.

In the turnover experiment, samples were measured using 90 min gradient optimized for each HpH fraction. Automatic gain control was set to “standard” and the mass spectrometer was operated in the positive ion mode. Full MS scans were acquired in a range of 300 to 1750 m/z at resolution of 60,000. Twenty most intense multiply charged ions were selected for HCD fragmentation with a dynamic exclusion period of 30 s and tandem MS (MS/MS) spectra were acquired at resolution of 30.000.

For all measurements of samples from microfluidic devices, peptides were eluted using a 60-min segmented gradient from to 10-33 to 50 to 90% of solvents A (0.1% formic acid) and B (80% acetonitrile in 0.1% formic acid) at a constant flow rate of 200 nl/min. The mass spectrometer was operated in the positive ion mode. Full MS scans were acquired in a range of 300 to 1750 m/z at resolution of 120,000. 20 most intense multiple-charged ions were selected for HCD fragmentation with a dynamic exclusion period of 30 s and MS/MS spectra were acquired at resolution of 15,000. Automatic gain control set to “custom”.

The column temperature was maintained at 40 °C using an integrated Sonation column oven. Peptides were ionized using nanospray ionization; the source temperature was set to 275 °C.

### MS Data Processing

The LC-MS/MS acquired DDA raw data was processed using the MaxQuant software package (version 2.2.0.0.). Spectra were searched against Uniprot *Homo sapiens* database (103,830 entries, downloaded 2022/12/16) and 286 commonly observed laboratory contaminants. The SILAC labels were defined as follows: Lys0 and Arg0 as the “light,” Lys4 and Arg6 as the “medium-heavy,” and Lys8 and Arg10 as the “heavy” channel. Carbamidomethylation on cysteine was set as a fixed modification, while N-terminal acetylation and methionine oxidation were selected as variable modifications. For MS and MS/MS, the peptide mass tolerance was set at 4.5 ppm and 20 ppm, respectively. Only two missed cleavages were allowed for the tryptic digestion. FDR was set to 1% at both peptide and protein level. Intensity-based absolute quantification (iBAQ) was enabled. For the turnover dataset, the “requantification” option was enabled.

For the DIA dataset, the MaxDIA version 2.4.2.0. was used in discovery mode, and the default parameters were used unless otherwise stated. Label-free quantification was enabled. In silico–predicted library for all human peptides with up to one missed cleavage was downloaded on 30.05.2023 from http://annotations.perseus-framework.org. The spectral libraries of peptides were uploaded to the MaxQuant software in the form of ‘evidence’ and ‘msms’ files. Carbamidomethylation on cysteine was set as a fixed modification, while N-terminal acetylation and methionine oxidation were selected as variable modifications. For MS and MS/MS, the peptide mass tolerance was set at 4.5 ppm and 20 ppm, respectively. Only two missed cleavages were allowed for the tryptic digestion. FDR was set to 1% at both peptide and protein level. Label-free quantification and iBAQ were enabled.

### MS Data Analysis and Statistical Analysis

Downstream analysis of the ‘proteinGroups.txt’ output table was performed in Perseus (version 1.6.15.0). Contaminants, “reversed” protein hits and proteins only identified by one site were filtered out. Proteins were functionally annotated with Gene Ontology (GO) Biological Processes, GO Cellular Compartment, GO Molecular Functions, and Kyoto Encyclopedia of Genes and Genomes, as well as MitoCarta3.0.

The fisher exact test (FDR ≤0.5) was used to assess the over-represented categories. It was performed with the PANTHER Classification System software (Version 18.0 released 2023-08-01), available online at https://www.pantherdb.org (53).

For the turnover experiment, normalized protein H/L ratios from proteinGroups.txt were used for protein quantification. Only proteins with ratios in four out of five time points in two out of three replicates were considered.

Label incorporation was calculated with the following formula:

(1) $$\frac{Heavy\;label\;intensity}{\sum label\;intensity\;}or\frac{Medium\;label\;intensity}{\sum label\;intensity\;}$$

Protein turnover rate (*k*) was determined using a described formula (54) independent of the growth rate (Equation 2), by linear regression of the natural logarithm of protein SILAC H/L ratio over time, where *m* is the number of time points (*ti*) and *rti* is protein H/L ratio measured in a time point *ti*. To determine the half-life of a protein (*T*1/2), the turnover rate *k* was divided by the natural logarithm (Equation 3).

(2) $$k=\frac{\sum_{i=1}^{m}\;\log_{e}\left(r_{t_{i}}+1\right)t_{i}}{\sum_{i=1}^{m}t_{i}^{2}}$$

(3) $$T_{1/2}=\frac{2\;}{k}$$

Protein turnover rates are reported in the Supplemental Table S2.

For the microfluidic experiment, label incorporation was calculated following Equation 1 from the intensities found in proteinGroups.txt.

For generation of Venn diagrams, the online tool https://www.stefanjol.nl/venny and https://bioinfogp.cnb.csic.es/tools/venny/index.html were used. Box plot analysis of label incorporation was prepared in the online tool: http://shiny.chemgrid.org/boxplotr/. Additional graphical visualization was performed in the R environment (version 4.1.1) and in GraphPad (version 8.0.1.). Statistical analysis was performed with two-tailed unpaired *t* test in GraphPad Prism. Significance is indicated by asterisks: ∗*p* ≤ 0.05, ∗∗*p* ≤ 0.01, ∗∗∗*p* < 0.001.

## Results

**Fig. 1:**
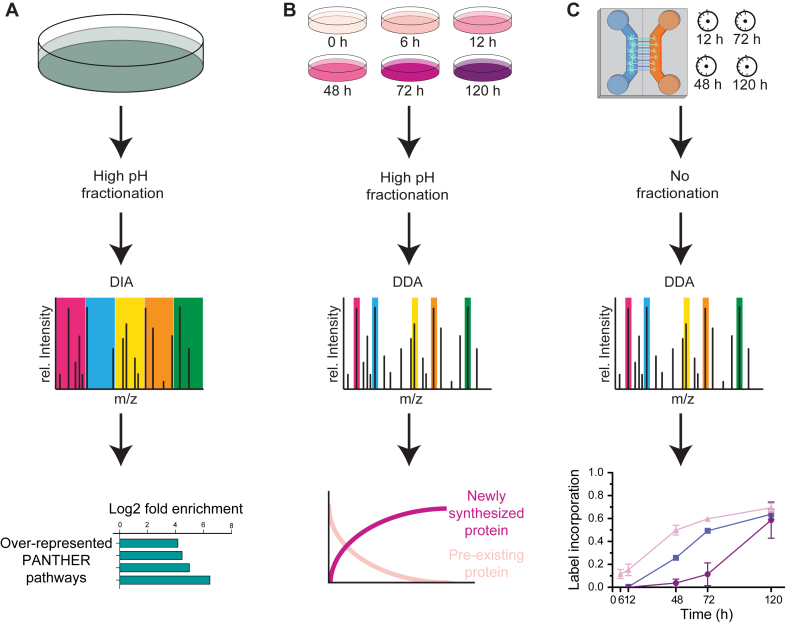
**Schematic representation of the methodologies employed for protein detection in hDANs.** The workflows delineate distinct procedures for protein quantification, turnover analysis, and investigation of local protein synthesis and trafficking. *A*, depiction of the first method, used to get a comprehensive coverage of the proteome of healthy hDaN cultures (day 23). *B*, schematic illustration showing an investigation of protein turnover dynamics using SILAC. Following cell differentiation and maturation (day 18), the culture medium is replaced with DMEM containing heavy (H) amino acids for varying durations (0 h, 6 h, 12 h, 48 h, 72 h, or 120 h) prior to protein harvesting at day 23 (n = 3 for each time point). *C*, schematic illustration of the dynamic SILAC approach applied to study local synthesis and trafficking of proteins in dopaminergic neurons. Following cell differentiation, cells are seeded in microfluidic devices, allowing axonal growth for 10 days. Subsequently, the culture medium is changed, with the soma well receiving DMEM containing medium-heavy (M) amino acids and the axonal well DMEM with heavy (H) amino acids for varying durations (12 h, 48 h, 72 h, or 120 h) prior to LC-MS analysis (n = 3 for each time point).

To perform a comprehensive analysis of proteome dynamics of hDaNs, we used three distinct workflows (Fig. 1). In order to maximize the proteome coverage, we first performed in-depth identification of proteins in hDaN cultures by high-pH fractionation followed by MS measurement using DIA. We next focused on the investigation of protein turnover using dynamic SILAC labeling of hDaNs under standard cell culture conditions. Finally, we analyzed differential expression, local synthesis, and trafficking of proteins between the axon and the soma by combining dynamic SILAC with culturing of neurons in microfluidic devices that featured physical separation of axons and soma. The hDaNs were differentiated from healthy human iPSCs using a previously described protocol (52) and characterization (13, 50, 51, 52, 55). Characterization of the markers of mature neuronal subtypes by microscopy (Supplemental Fig. S5, *A* and *B*) and mass spectrometry (Supplemental Fig. S5*C*) confirmed the dopaminergic lineage.

### The Proteome of iPSC-Derived hDaNs to a Depth of 9409 Proteins

Healthy hDaNs were bulk-harvested at day 23 from three independent hDaN differentiations. After trypsin digestion, peptides were fractionated using high pH reversed-phase chromatography and measured by DIA on an Orbitrap Exploris 480 instrument. This resulted in the identification of 9409 protein groups. Based on the coverage of annotated mitochondrial proteins (913 or 80.6%) and mitochondrial outer membrane proteins (91 or 81.3%), we estimate similar coverage of the cellular proteome (Supplemental Fig. S1, *A* and *B* and Supplemental Table S1-1). Identified proteins were stratified according to their intensity into five bins (Supplemental Fig. S1*C*); the Fisher exact test (FDR< 0.05) was performed to determine which molecular pathways, according to Panther (53, 56), were enriched in each intensity bin (Supplemental Fig. S1*D* and Supplemental Table S1-2). Within the bin with the highest DIA intensity, we detected a significant over-representation of proteins involved in Huntington’s disease, Parkinson’s disease, and Cytoskeletal regulation by Rho GTPase and glycolysis. They were followed by proteins from central metabolic pathways such as TCA cycle, pyruvate metabolism, pentose phosphate pathway, and glycolysis in the bin with the second-highest DIA intensity. Interestingly, physiological processes of dopaminergic neurons including those related to PD such as the ubiquitin-proteasome pathway, synaptic vesicle trafficking, Hedgehog signaling, dopamine receptor signaling, and axon guidance were also enriched in this bin (Supplemental Fig. S1*D*).

### Dynamic SILAC Enables Measurement of Protein Turnover for over 4300 Proteins

**Fig. 2:**
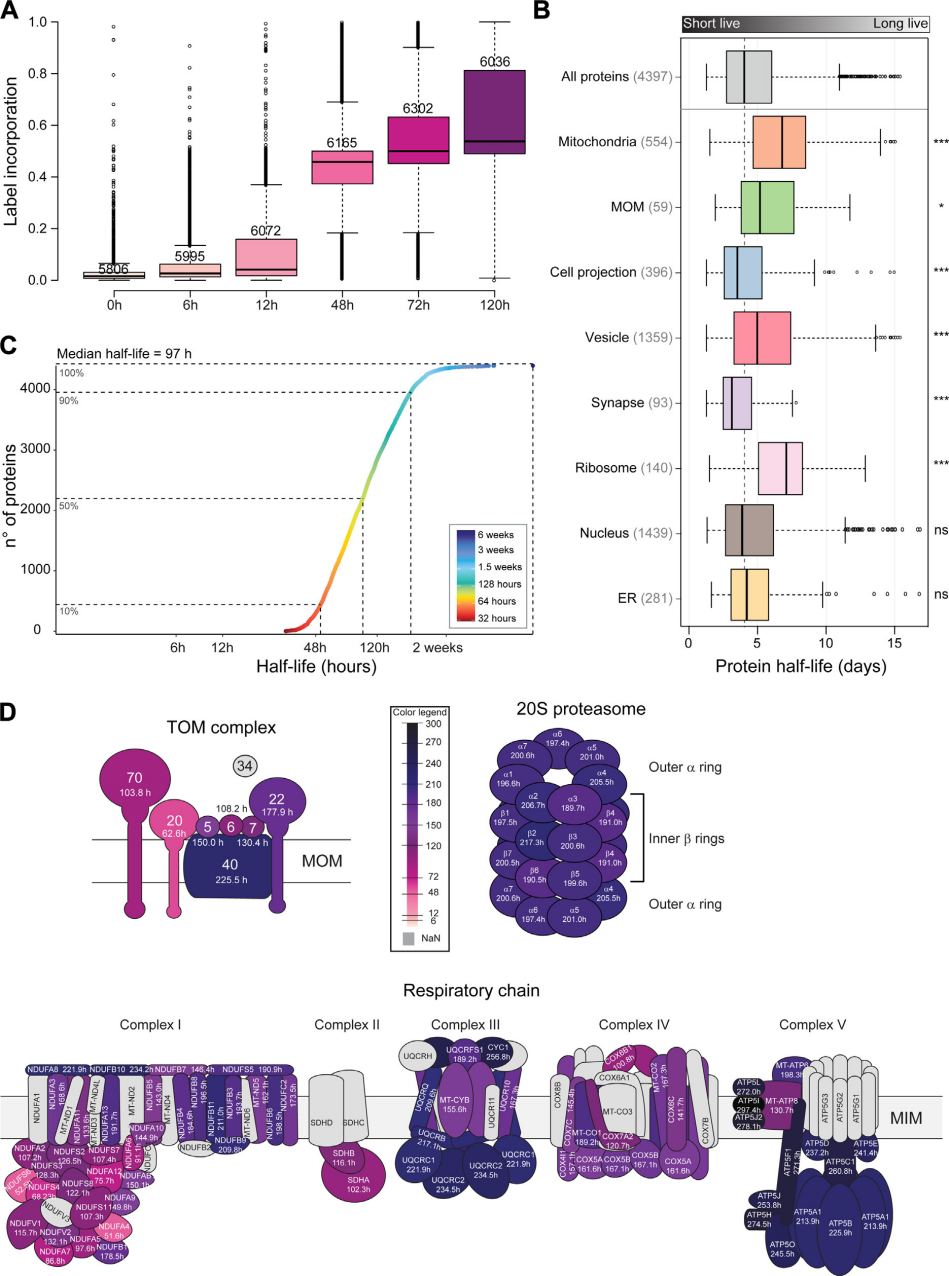
**Protein turnover of hDANs.***A*, SILAC label incorporation across all time points calculated as H/(L + H). Only proteins with ratio in two out of three replicates were considered. Number of proteins depicted are n = 5806, 5995, 6072, 6165, 6302, 6036 for their corresponding time points. *B*, distribution of total proteins with measured half-lives (4397) organized in eight different cellular compartments according to their GOCC annotation. Number of protein groups assigned to each compartment is given in brackets. Significant ∗*p* ≤ 0.05, ∗∗*p* ≤ 0.01, and ∗∗∗*p* ≤ 0.001, *t* test. For the boxplots, center lines show the medians; box limits indicate the 25th and 75th percentiles as determined by R software; whiskers extend 1.5 times the interquartile range from the 25th and 75th percentiles, outliers are represented by dots. *C*, range of log_2_ scale half-lives of hDANs proteins, with a median of 97 h. *D*, schematic illustration of the TOM complex (*upper left* panel), the 20S proteasome (*upper right* panel), and the respiratory chain (*lower* panel) showing half-lives for individual components of each complex. Proteins are color coded as a gradient from *light pink* (shortest half-life) to *dark blue*-*black* (longest half-life).

In order to generate a reference protein turnover dataset, we pulsed differentiated dopaminergic neurons with “heavy” stable isotope-labeled Arg and Lys amino acids and harvested the cells after five time points (6, 12, 48, 72, and 120 h). Following high-pH reversed-phase fractionation and MS measurement, about 6000 protein groups were quantified in each time point (Supplemental Table S2-1). The average label incorporation after 120 h was approximately 50% (Fig. 2*A* and Supplemental Table S2-2). We next used the previously described approach to calculate protein half-lives (54), taking into consideration only proteins with detectable label incorporation in at least four out of five time points in at least two out of three replicates. This led to determination of the protein half-life for 4397 proteins (Supplemental Table S2-3), out of which 554 were annotated as mitochondrial and 59 as mitochondrial outer membrane proteins. The measured half-lives ranged from 1 day to more than 20 days (Supplemental Fig. S2*B*), with a median of 97 h (Fig. 2*C*).

We then examined the relationship between protein half-life and its cellular location and/or function. As depicted in Figure 2*B*, most proteins exhibited a diverse range of half-lives that were not significantly different from the population average. However, in agreement with previous reports, both mitochondrial and ribosomal proteins deviated from the population average and exhibited significantly longer half-lives than other groups. Synaptic proteins, on the other hand, had significantly shorter half-lives than the median of all proteins. We next stratified proteins according to their half-life into seven bins (Supplemental Fig. S2*C*), subjected each bin to a Fisher’s exact test (FDR< 0.05), and generated over-represented pathways according to the Panther database (Supplemental Fig. S2*D* and Supplemental Table S2-4). Among the most short-lived proteins (less than 3 days), synaptic vesicle trafficking, Wnt signaling, and Alzheimer’s disease-amyloid and -presenilin pathways were over-represented. Multiple signaling pathways, such as purine synthesis, pyruvate metabolism, TCA cycle, cholesterol metabolism, and glycolysis, were highly enriched in a group with longer half-lives (between seven and 11 days). Among the proteins with average half-lives (between 3 and 7 days), dopamine receptor-mediated pathways, DNA replication, hormone signaling, and PDGF signaling were enriched (Supplemental Fig. S2*D*). We next focused on protein complexes and pathways. Whereas conserved complexes and protein classes, such as the proteasome subunits, E3-ligases, and Rab proteins, had relatively uniform half-lives, we observed several interesting outliers. In agreement with previous reports, the components TOM20 and TOM70 of the TOM complex had shorter half-lives than other TOM components (Fig. 2*D* and Supplemental Fig. S3*A*). Interestingly, the peptidyl-prolyl cis-trans isomerase FKBP8, a putative chaperone that plays a role in mitophagy and apoptosis, had a significantly shorter half-life than other members of these pathways, making it an interesting subject for further investigations (Supplemental Fig. S3*C*). Also interesting were the proteins from the cytosolic (80S) ribosome, which had significantly different half-lives between large and small ribosomal subunit (Supplemental Fig. S3, *A* and *B*). This was not the case for the proteins of the mitochondrial (70S) ribosome, which overall had shorter and more uniform half-lives than the 80S-proteins (Supplemental Fig. S3, *A* and *B*). We then investigated the turnover rates of respiratory chain complexes due to their association with mitochondrial dysfunction in Parkinson’s disease. There is a large range of turnover across complexes and subunits, especially within complex I (Fig. 2*D*), which contains the most subunits (44 in total, including seven encoded by the mitochondrial DNA). Complex V is a large complex and contains subunits that have the longest half-lives (notably, none of the alpha subunits of complex V were detected), while the peripheral arm of complex I has the shortest half-lives (Fig. 2*D*). We compared the respiratory complex turnover rates from dopaminergic neurons used in this study (post-mitotic), with published data from cancer cells, which are proliferative (57), and found that overall respiratory chain complex turnover is slower in dopaminergic neurons than cancer cells (Supplemental Table S2-6). Interestingly, slower turnover of respiratory complex subunits in dopaminergic neurons as compared to cancer cells was not observed for those subunits encoded by the mitochondrial genome (Supplemental Table S2-6).

### Microfluidic Devices Enable Differential Analysis of Somatodendritic and Axonal Proteins

In order to study proteome dynamics in axons, we used two-well microfluidic devices (Xona Microfluidics), which feature fluidic isolation between the wells. In such devices, differentiating neurons seeded in one well (termed proximal or somatodendritic well) elongate their axons across the 900 μm-long microgroove barrier into the adjacent well (termed distal or axonal well). This allows for a spatial differential analysis of the somatodendritic and axonal proteome and selective usage of the SILAC label in the somatodendritic or in the axonal well.

**Fig. 3:**
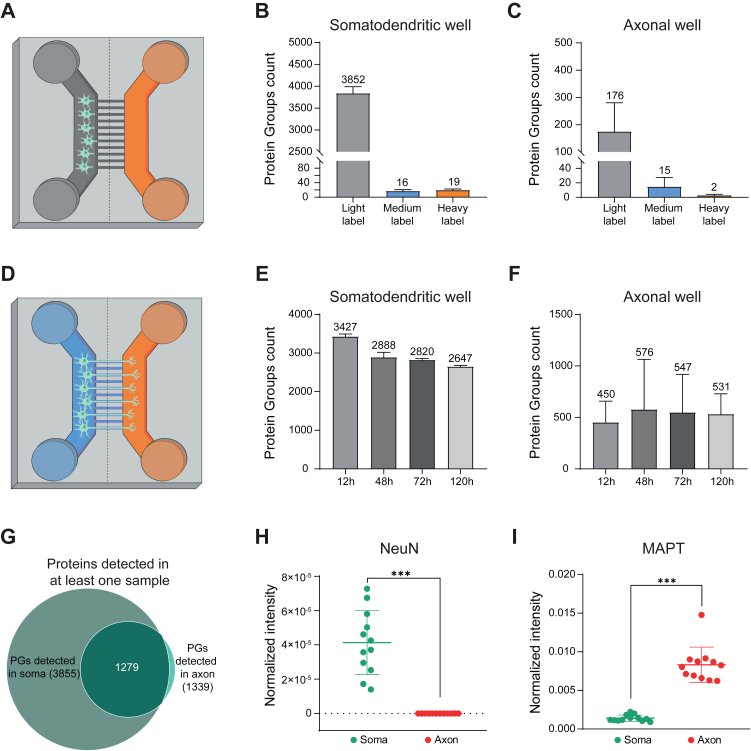
**Qualitative proteome analysis of hDANs cultured in microfluidic devices.***A*, schematic illustration of the experimental set up of diffusion experiment. hDANs were seeded in the proximal well (*gray* color) and heavy SILAC medium was added into the empty distal well (*orange* color) after 24 h. *B*, number of PGs with each label identified in the soma sample in diffusion experiment after incubation for 48 h. *C*, number of PGs identified with each label in the axon sample in diffusion experiment after incubation for 48 h. *D*, schematic illustration of the experimental set up and identifications in time course experiment. hDaNs were seeded into the somatodendritic well (*blue* color) and allowed for axonal elongation into the axonal well (*orange*). After 10 days, the medium-heavy and heavy label were added into the somatodendritic well and the axonal well, respectively. *E*, number of PGs identified in each time point in the somatodendritic well. *F*, number of PGs identified in each time point in the axonal well. All the bar graphs represent mean with error bars (SEM) from three biological replicates. *G*, Venn diagram of identified PGs in at least one sample in the soma side (3855 in total) and in the axon side (1339 in total). *H*, normalized intensity of NeuN, a known soma marker, from all time points and replicates from somatodendritic well (*green*) and the axonal well (*red*) are shown. *I*, normalized intensity of MAPT, a known axonal marker, from all times points and replicates from somatodendritic well (*green*) and the axonal well (*red*) are shown. ∗∗∗*p* ≤ 0.001, *t* test. PG, protein group.

To validate the absence of fluidic diffusion between the wells, we first seeded neurons (day 9 post-differentiation start) in the proximal well, and 24 h after seeding, added the heavy SILAC medium into the empty distal well. This timeframe was long enough to allow cell attachment but too short for axons to protrude through the microgroove barrier (Supplemental Fig. S4*A*). After 48 h of SILAC incubation, only 0.02% of the proteins in the proximal well were detected with the heavy label, which was below the FDR threshold of 1%. As a negative control, we also searched the database for medium-heavy labeled proteins, which were not present in the sample. As expected, the number of identified medium-heavy labeled proteins was below the 1% FDR threshold (Fig. 3, *A*–*C*), validating the dataset. This experiment confirmed the absence of detectable fluidic diffusion of heavy amino acids between the wells. We also performed live imaging of the microfluidic devices over a 24 h period where we followed mitochondria stained in the axonal side only by using MitoTracker deep red and neutral lipids stained in the soma side only by using BODIPY-green (Supplemental Fig. S5, *D*–*F*). This experiment confirmed only intracellular transport of stained mitochondria or lipids at the microgroove barrier.

To independently investigate the proteome dynamics of somatodendritic and axonal proteins, we seeded the differentiating hDaNs into the proximal (somatodendritic) well and allowed for axonal elongation across the microgroove barrier into the distal (axonal) well (Supplemental Fig. S4*B*). After 10 days, we added different SILAC amino acids into each well: medium-heavy (Lys4/Arg6) in the somatodendritic well and heavy (Lys8/Arg10) in the axonal well (Fig. 3*D*). The employed combination of labeled amino acids allowed us to determine the origin of each detected protein and therefore address local protein synthesis and transport between axons and the soma. Samples from both wells were harvested at 12, 48, 72, and 120 h after SILAC labeling and measured separately by LC-MS. This led to quantification of up to 3400 protein groups in the somatodendritic well and about 500 proteins in the axonal well across all timepoints (Fig. 3, *E* and *F* and Supplemental Table S3-1).

We next assessed the overlap of proteins identified in the somatodendritic and axonal well. Of a total of 3915 proteins, 1279 were detected in both wells, with 60 exclusively detected in the axonal well and 2576 in the somatodendritic well (Fig. 3*G*). Principal component analysis performed between the somatodendritic and axonal proteins revealed two separate, nonintersecting clusters (Supplemental Fig. S4*C*). To assess the purity of each well, we compared normalized intensities of several marker proteins. Neuronal nuclear protein NeuN, present exclusively in the nucleus (soma), was significantly enriched in samples from the somatodendritic well (Fig. 3*H* and Supplemental Table S3-3), whereas proteins MAPT and MAP1B, known structural and axonal markers, were enriched in samples from the axonal well (Fig. 3*I* and Supplemental Fig. S4*D* and Supplemental Table S3-3).

We further investigated the 60 proteins exclusively detected in the axonal well, as well as those with four-fold higher relative abundance in the axonal well than the somatodendritic well (total of 67 proteins) (Supplemental Fig. S4*E*). This combined dataset of 127 proteins included 22 proteins classified as axonal according to GO, among which were the known axonal markers MAPT and MAP1B. The remaining 105 proteins have a likely axonal localization but have not yet been annotated as axonal proteins (Supplemental Fig. S4*F* and Supplemental Table S3-4). We validated the axonal location of two of these proteins using immunofluorescence microscopy, namely GAP43 and STMN2 which had a high fluorescence intensity in the axons (Supplemental Fig. S5, *G* and *H*). We next subjected the list of proteins exclusively detected in the axonal well and those enriched in axons to a Fisher exact test (FDR< 0.05). Cell-cell contact zones, synaptic vesicle membrane, synaptic vesicle, SNARE complex, and exocytic vesicles were highly enriched among other Panther terms, further pointing to axonal specificity (Supplemental Fig. S5*G* and Supplemental Table S3-5).

### Differential SILAC Labeling Reveals Local Protein Synthesis and Transport Between the Soma and Axons

**Fig. 4:**
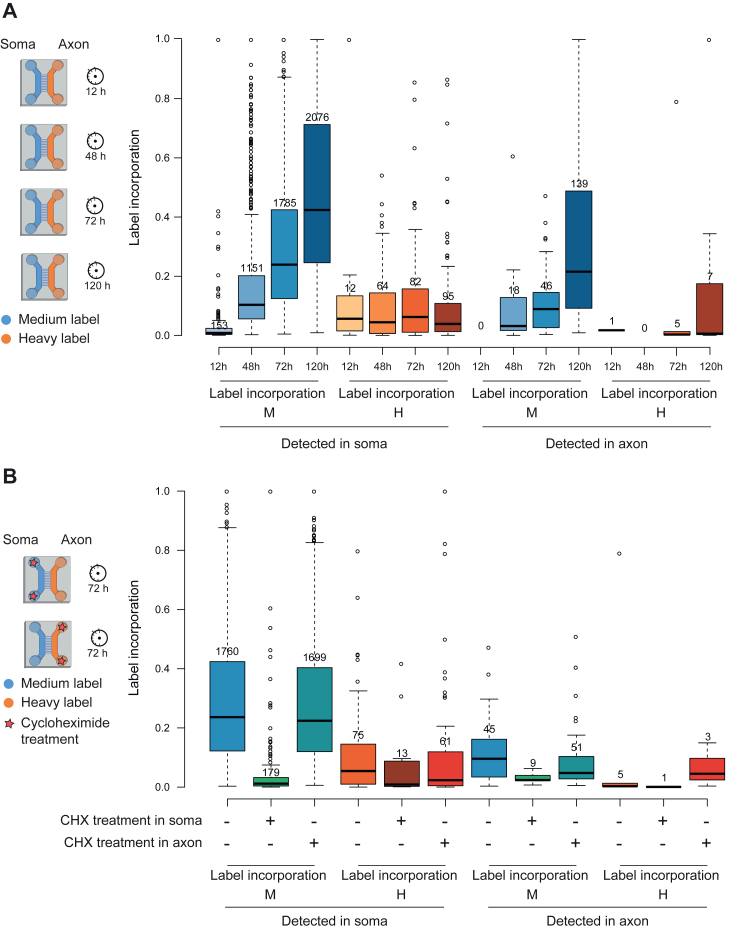
**Quantitative proteome analysis of hDANs cultured in microfluidic devices**. *A*, schematic illustration of the experimental set up (*right* label) for time course experiment. Boxplots depicting SILAC label incorporation (*left* panel) across all time points in the soma well and the axon well calculated as H/(L +M+ H) and M/(L +M+ H). Only those proteins with label incorporation in two out of three replicates were considered. Color coding: *blue* for label in the soma side and *orange* for label in the axon side, with increasing darkness for longer time points. *B*, schematic illustration of the experimental set up (*right* label) for protein synthesis inhibition by CHX experiment. Boxplots depicting label incorporation (*left* panel) across all samples in the soma side and the axon side calculated as H/(L +M+ H) and M/(L +M+ H). Only those proteins with label incorporation in two out of three replicates were considered. Color coding: *blue* tone for soma side label, *orange* tone for the axon side label. For the boxplots, center lines show the medians; box limits indicate the 25th and 75th percentiles as determined by R software; whiskers extend 1.5 times the interquartile range from the 25th and 75th percentiles; outliers are represented by dots.

We next analyzed the SILAC label incorporation in each well. Incorporation of the medium-heavy SILAC label in proteins detected in the somatodendritic well (Fig. 4*A* and Supplemental Table S3-2) followed a pattern very similar to that of whole neurons (Fig. 2*A*). This was expected, as the cells in this well are very similar to the neuronal cell (not all axons generated by the neurons in the somatodendritic well cross the microgroove barrier). Importantly, 141 proteins detected in the axonal well contained the medium-heavy SILAC label, pointing to their synthesis in the soma and anterograde transport into the axons. Conversely, 154 proteins detected in the somatodendritic well contained the heavy SILAC label, pointing to their synthesis in the axon and retrograde transport into the soma. Of note, 107 out of these 154 proteins were also detected in the unlabeled axonal proteome (Fig. 3*G*). In total, differential SILAC labeling revealed 269 proteins that were trafficking between the soma and the axon during the time window of our analysis (26 of them in both directions).

To validate local protein synthesis, we inhibited translation by adding CHX together with the SILAC labels for 72 h in each well separately. As expected, the CHX treatment in the somatodendritic well almost completely abolished incorporation of the medium-heavy SILAC label, pointing to a block in protein synthesis. Interestingly, CHX treatment in the somatodendritic well also decreased incorporation of the heavy SILAC label in the axonal well. CHX treatment in the axonal well did not lead to any detectable effect, likely due to the low amount of proteins detected in the axonal well (Fig. 4*B*, Supplemental Tables S3-7, and S3-8). Combined, these results confirmed that dynamic SILAC applied to microfluidic devices can measure protein synthesis independently in the soma and the axon.

**Fig. 5:**
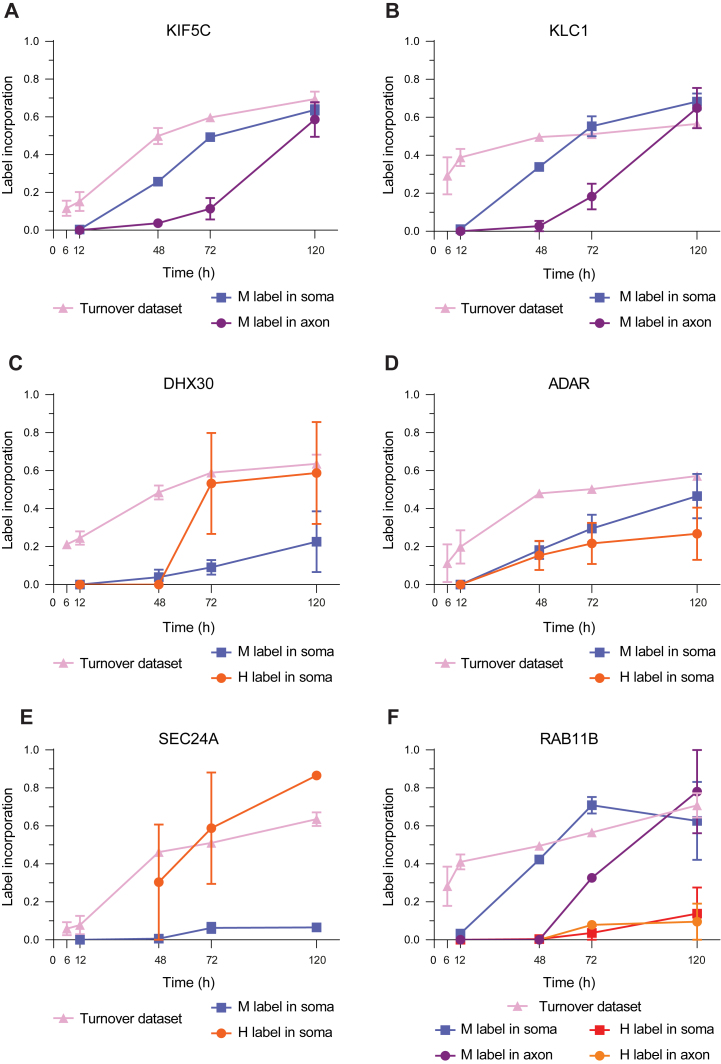
**Examples of proteins trafficked between soma and axon.***A*, SILAC label incorporation curves of the medium-heavy label of KIF5C in the soma side and in the axon side and the label incorporation curve of KIF5C in the turnover dataset. *B*, label incorporation curves of the medium-heavy label of KLC1 in the soma side and in the axon side and the label incorporation curve of KLC1 in the turnover dataset. *C*, label incorporation curves of the heavy and medium-heavy label of DHX30 in the soma side and the label incorporation curve of DHX30 in the turnover dataset. *D*, label incorporation curves of the heavy and medium-heavy label of ADAR in the soma and the label incorporation curve of ADAR in the turnover dataset. *E*, label incorporation curves of the heavy and medium-heavy label of SEC24A in the soma side and the label incorporation curve of SEC24A in the turnover dataset. *F*, label incorporation curves of the heavy and medium-heavy label of RAB11B in the soma side and in the axon side and the label incorporation curve of RAB11B in the turnover dataset.

We next focused on the protein transport between the soma and the axon. To this end, we analyzed temporal SILAC incorporation profiles of proteins known to be trafficking between the two cellular components. We focused on the Kinesin Family Member 5C (KIF5C), a motor protein essential for intracellular transport, primarily involved in anterograde transport, moving cargo from the neuron cell body (soma) towards the synaptic terminals in the axon (58). In our dataset, KIF5C was labeled with medium-heavy SILAC and detected both in the soma sample and in the axonal well (Fig. 5*A*). Importantly, the patterns of the SILAC label incorporation were markedly different in the soma and in the axon: the pattern in the soma was similar to that measured in the whole neuronal cell, where 50% of the protein was labeled after 72 h. However, in the axon, only 10% of the protein was labeled in that time frame. Interestingly, after 120 h, the label incorporation was identical in both soma and axon, pointing to the time frame needed for the pool of newly synthesized KIF5C molecules to equilibrate within the cell. Upon CHX treatment in the soma, the protein was no longer synthesized (Supplemental Fig. S6*A*).

Kinesin Light Chain 1 (KLC1) is an integral part of the kinesin-1 motor complex, since this complex is typically formed by two kinesin heavy chains (KIF5s) and two KLCs (59). Similar to KIF5C, the curve of medium-heavy label incorporation in the soma and in the axon were different. In this case, after 72 h, 55% of KIF5C was labeled in the soma, but only 15% was labeled in the axon (Fig. 5*B*). Upon CHX treatment in the soma, the protein was no longer newly synthesized (Supplemental Fig. S6*B*).

Interestingly, several proteins that are involved in RNA biology were detected as synthesized in the axon and transported into the soma. These included adenosine deaminase ADAR1 (double-stranded RNA-specific adenosine deaminase) (60) and RNA helicase DHX30 (ATP-dependent RNA helicase DHX30), involved in the assembly of mitochondrial ribosomes (61). Both proteins were labeled with medium-heavy SILAC and with heavy SILAC and detected in the soma sample. The heavy labeled DHX30 increased in the soma at a faster rate than the medium-heavy labeled, which highlights the importance of the synthesis of this protein in the axon and its retrograde transport (Fig. 5*C*). Overall, the majority of the DHX30 protein copies in the soma were synthesized in the axon. On the other hand, ADAR1 is also synthesized in the axons and later on transported towards the soma, but at a slower rate (Fig. 5*D*). We validated the localization of DHX30 using fluorescence microscopy, which was mainly localized in the axons (Supplemental Fig. S5*I*).

Another group of interesting trafficked proteins are those involved in vesicle transport such as SEC24A (Protein transport protein Sec24A) and RAB11B (Ras-related protein Rab-11B). SEC24A is a component of the coat protein complex II, which plays a crucial role in the formation of transport vesicles from the endoplasmic reticulum (ER). It was labeled with medium-heavy SILAC and with heavy SILAC. Similar to DHX30, the amount of heavy label SEC24A was higher than the medium-heavy label, which suggests that the majority of the protein in the soma was synthesized in axon and retrogradely transported (Fig. 5*E*). RAB11B belongs to the Ras superfamily of small GTPases, which are key regulators of intracellular membrane trafficking, from the formation of transport vesicles to their fusion with membranes (62). RAB11B was labeled with medium-heavy SILAC and detected both in the soma sample and in the axonal sample and was also labeled with heavy SILAC and detected both in the soma sample and in the axonal sample. The label incorporation indicated that the majority of the protein was synthesized in the soma and transported to the axonal side. However, some of the RAB11B was also synthesized in the axon and transported to the soma (Fig. 5*F*).

## Discussion

Using previously characterized iPSC-derived hDaNs, we report the proteome, protein turnover and the first spatial proteome datasets for this cell type and model system. Our study provides a workflow and resource for future applications of quantitative proteomics to cultured human neurons in general.

We first confirmed the dopaminergic differentiation of neurons using immunofluorescence-based and proteomic-based characterization of mature neuronal markers (Supplemental Fig. S5, *A* and *B*). We detected serotonergic marker TPH2 as the second most abundant mature neuronal marker (Supplemental Fig. S5*C*), indicating that our cultures contained serotonergic neurons. We did not detect significant markers of glutaminergic or GABAergic neurons (Supplemental Fig. S5*C*). This is in line with previously published characterization of the differentiation method (13, 51, 55).

We next employed extensive fractionation and DIA to achieve high proteome coverage. We identified over 9400 proteins from bulk extraction of iPSC-derived hDaNs. The coverage of the mitochondrial and mitochondrial outer membrane proteomes was >80% based on MitoCarta 3.0, one of the best annotated reference proteomes available to date (63). Mitochondrial proteins and their dynamics have a pivotal role in dopaminergic neurons and in neuronal health, linking them to energy metabolism and the pathophysiology of neurodegenerative disorders (64, 65, 66). Previous studies have relied on prior enrichment of mitochondria to achieve 65% coverage using DIA for iPSC-derived hDaNs (13) and in mapping more than 1100 mitochondrial proteins in mammalian cells (57). Since we report the first axonal proteome for human iPSC-derived dopaminergic neurons, we compared our data to two existing axonal transcriptomes from human iPSC-derived spinal motor neurons and mouse primary neurons. The first study with spinal motor neurons used the same devices (67) and we found an overlap of nine gene/protein groups. In the other study, we found an overlap of 37 gene/protein groups where another microfluidic system utilizing mouse neurons was used (68). The relatively low overlap may be attributed to different cell types and technological platform (transcriptomics) used in these studies.

Our study also provides the first protein turnover dataset for iPSC-derived hDaNs. Most of the studies that addressed proteome turnover data to date have employed tissue or primary neuronal cultures from rodents (24, 26) or mRNA data from human brain tissue (69), nonmammalian cells (70), or human midbrain-like organoids (71). These studies led to the discovery of cell type specificity and influence of glia and other non-neuronal subtypes on the neuronal proteome. Pathway analysis based on our protein turnover data showed that the longest-lived proteins associated with central metabolism; pyruvate metabolism and *de novo* purine and cholesterol biosynthesis, biosynthesis of nucleotides, and the ubiquitin proteasome pathway. We also detected serotonergic pathways such as 5-HT degradation together with PDGF, EGF, and VEGF signaling, confirming the presence of serotonergic neurons and possibly oligodendroglia and neuroepithelial cells.

Depiction of the half-lives of individual subunits of the proteasome highlights the relatively long lived, uniform, synchronous turnover of the proteasome in hDaNs. In contrast, a study using mouse embryonic neurons reported shorter half-lives of those that we observe (19). Previous studies have reported an inverse correlation between organismal life-span and protein turnover (72, 73). This may explain why mouse embryonic neurons have shorter protein half-lives than human neurons.

In our study, long-lived processes included cytoskeletal regulation by Rho GTPases, which include a large number of proteins involved in cytoskeletal processes crucial for neuronal function such as the filopodia, lamellipodia, and vesicle transport. The mitochondrial Rho proteins regulate several aspects of mitochondria homeostasis and transport. One of the encoded proteins, a Rho GTPase known as Miro1, was detected in two protein groups and had a half-life of 99.8 h for isoforms 1/4 and 126.9 h for isoforms 3/5/7 in hDaNs. Miro1 regulates mitophagy and calcium handling and is currently a biomarker and drug development target for PD (74, 75). We also looked in more detail at mitophagy and the TOM complex. Component subunits of the TOM complex have non-uniform half-lives, in agreement with a previous report (57). The detailed organization and binding activities of the TOM complex are particularly relevant in PD, since the interaction of PD protein PINK1 with the TOM complex is intensely studied due to its relevance for activation of the kinase (76, 77, 78, 79). Similar to previous studies in rat primary neuron cultures, we show that synaptic proteins tend to be short-lived, while mitochondrial proteins tend to be long-lived (25, 26). We further looked into the half-lives of ribosomal proteins. Our analysis revealed distinctions in the stability of proteins associated with the large and small subunits of the ribosome (Supplemental Fig. S3, *A* and *B*). Interestingly, the 55S mitochondrial ribosome proteins had significantly shorter half-lives than their 80S human counterparts, with no discernible variations in protein half-lives between its two subunits (Supplemental Fig. S3, *A* and *B*). The mitochondrial ribosomes are subjected to substantial oxidative damage, particularly in neurons, which could explain the need for a faster turnover. To our knowledge, this was never reported so far. However, it should be mentioned that the coverage of the cytosolic (80S) ribosome proteins in our dataset was 94%, while the coverage of the mitochondrial (70S) ribosome was 70%, so there is a chance that some proteins with lower turnover were not detected.

Most axonal proteins in neurons are delivered from their site of synthesis in the soma to the axon *via* anterograde vesicular transport and undergo retrograde transport for redistribution and/or lysosomal degradation (80). After synthesis in the cell body, proteins are transported down the axon as various kinds of membrane organelles or protein complexes (81). Furthermore, active transport is the primary mechanism by which organelles, proteins, nucleic acids, and lipids are delivered to relatively distant regions of a growing neuron (82). To address local protein synthesis and transport between axon and soma, we developed and optimized hDaN spatial proteomics using microfluidic devices. This is particularly challenging, due to the very small amount of starting material that can be harvested from the chambers for mass spectrometry. Other groups have successfully performed axon *versus* soma analysis from mouse brain slices with apex (18) and the nascent proteome in retinal axons from *Xenopus laevis* using dynamic SILAC (41). Here we provide the first evidence of local protein synthesis of 154 proteins in the axon and their retrograde transport to the soma of hDaN. In addition, we provide a database for proteome dynamics (axons *versus* soma) in hDaNs over a 120 h period. We did not identify retrograde transport of proteins previously shown to be synthesized in mouse axons and transported to the soma such as Importin, Stat3, mTOR, CREB, or ATF4 following injury (45, 46, 47, 48, 49). These protein examples are not ideal controls because in our study, we do not induce axonal injury. Instead, we chose six other proteins as examples to demonstrate and discuss the method: KIF5C, KLC1, DHX30, ADAR, SEC24A, and RAB11B.

There are three isoforms of Kinesin Family Member 5 (KIF5), the ubiquitous KIF5A, KIF5B, and KIF5C, which are isoforms found specifically in neurons (83). Interestingly, KIF5C was the only isoform of KIF5 detected to be newly synthesized and transported within the 120 h time period. It was also the most abundant of all three forms, while KIF5A was not detected at all time points (Supplemental Fig. S7*B*). The half-life of KIF5B was 116 h, while of KIF5C is 68 h, which could also explain the lack of observed label incorporation seen for KIF5B in the time course of our study (Supplemental Fig. S7*A*). Interestingly, KIF5 motors bind Miro1 to facilitate mitochondrial trafficking and importantly, mitochondrial stopping (84), a prerequisite for mitophagy.

KLC1 had similar dynamics to KIF5C. Transcriptome analysis in mice revealed that KLC1 splicing could modify amyloid beta accumulation (85) and therefore could contribute to AD. Later studies showed that KIF and KLC1 implicated axoplasmic transport is disrupted in AD and axonal transport in general is significantly associated with neurodegenerative diseases (86). Another protein of the transport machinery, DYNC1H1 (Dynein, cytoplasmic 1, heavy chain 1), is a component of the cytoplasmic dynein complex, primarily known for its role in retrograde transport, moving cargoes from the axon terminal back to the soma (59). We observed it to be newly synthesized in the soma but could not detect it with medium-heavy label in the axon. Notably, it was also detected as heavy-labeled in the soma, which indicated that it is also synthesized in the axon (Supplemental Fig. S7*C*) and CHX treatment abolished its synthesis in the soma (Supplemental Fig. S7*D*).

Since the length of the microgroove barrier is known (900 μm), as are the axonal velocity rates of kinesin and dynein (0.5–1.0 μm/sec) (87), it is tempting to analyze our data in the context of transport rates. However, these rates will be difficult to estimate, as we measure the bulk protein population in numerous cells and our approach requires extra time to accumulate enough protein molecules to detect the signal on each side of the barrier.

An increasing number of studies has highlighted the crucial role of local protein synthesis in axons. In particular, the role of local synthesis of mitochondrial proteins in axons to maintain the health of neurons and meet energy demands (88, 89). Interestingly, the mRNA population only partially predicts the local protein population in neurons and this relationship significantly varies between different gene groups (90). Our method could assist in better dissection of RNA transport and local protein synthesis in iPSC-derived neurons. Of 154 proteins that incorporated the heavy SILAC label and were therefore synthesized in the axon, we discuss only those with the highest label incorporation.

DHX30 is an RNA helicase that is part of the DExD/H-box protein family, which is involved in various aspects of RNA metabolism. It has been shown to be a RNA granule protein and has an important role in the assembly of mitochondrial ribosomes (61). In our study, this protein was synthesized locally in the axons and transported in a retrograde manner into the soma. Another interesting protein in terms of dynamics was ADAR, which catalyzes the hydrolytic deamination of adenosine to inosine in dsRNA, which is referred to as A-to-I RNA editing (60). In our dataset, we refer to ADAR1, which is an essential player in the regulation of cellular immune responses, transcriptomic diversity, and cell senescence (91). Of a small number of conserved mammalian ADAR editing sites, they tend to be located in genes encoding neurotransmitter receptors or other synapse-related proteins (92). Indeed, this protein is reported to have an important role in metazoan nervous system, where it modifies pre-mRNAs of proteins involved in electrical and chemical neurotransmission, such as pre-synaptic release complexes and voltage- and ligand-gated ion channels (93). So far, it was reported to be localized mainly in the soma (nucleus) (93). The proteomics data points to the importance of the synthesis of this protein in the axon and its retrograde transport into the soma. Finally, we focused on the protein transport protein SEC24A, a component of the coat protein complex II and RAB11B, a Rab family GTPase involved in the transport of vesicles and endocytic recycling important for synaptic function. SEC24A regulates the control of the formation of transport vesicles from the ER and Ca^2+^ flux between the ER and mitochondria (94). In our dataset, the majority of SEC24A was synthesized in the axons and retrogradely transported to the soma after 48 h. Rab proteins have been associated with PD following discovery of Rab8A, Rab10, Rab8A, Rab8B, and Rab13 as substrates of the PD kinases LRRK2 (95) and PINK1 (96). In our study, we detected Rab11B, which is highly expressed in the brain and involved in recycling *via* the recycling endosome (97) and implicated in several neurodegenerative diseases (98). Rab11 is particularly interesting in the context of mitochondrial dysfunction in PD since many Rab proteins are essential in regulating autophagy and Rab11 was shown to regulate mitophagy downstream of PINK1 and parkin (99) and upstream of alpha synuclein (100). In hDaNs, Rab11B was synthesized in both soma and axons and transported bidirectionally.

### Limitations of the Study

Studying global protein turnover and transport in cultured iPSC-derived hDaNs is challenging, and our study is not free of experimental bias. Dynamic SILAC is a powerful method to study protein turnover, but its application over a relatively short experimental time frame leads to low label incorporation in proteins with low turnover, which in turn leads to high variability of the measured SILAC ratios. Conversely, high turnover proteins may already have been completely labeled by the time the first sample was taken for analysis (6 h). It is therefore important to note that half-lives of proteins with high or low turnover are either missing or are prone to high measurement error due to the employed experimental design. Furthermore, recycling of unlabeled amino acids released from the degradation of pre-existing proteins may cause a dilution of the labeled amino acid pool and result in apparently lower turnover rates, as described before (101). These values can be corrected using a recycling factor that can be calculated by measuring label incorporation in partially labeled missed cleaved peptides (102). Although these issues may influence the overall sensitivity and accurate estimation of individual protein turnover rates, we do not expect that they lead to a false detection of the newly synthesized proteins in our dataset.

Culturing of neurons in Xona microfluidic devices, as done in this and other studies (103), provides a robust way of separating axons and soma. However, the relatively small well size enables seeding of only up to 140,000 cells in the proximal (somatodendritic well), which leads to a very low amount of proteins extracted from the distal (axonal) well. Indeed, based on the median of the total iBAQ intensity in all samples, we estimate that the biomass in the distal well was about 33 times lower than in the proximal well. The low number and low intensity of identified proteins in the axonal well pose a significant challenge for quantitation, due to the dynamic range needed to measure low SILAC incorporation. For this reason, heavy-labeled proteins (synthesized in axon) were mainly detected in the somatodendritic well, where the protein amount and signal intensity were higher. Given that the amount of cell seeding is limited by the employed microfluidic devices, increasing cell seeding would lead to the clogging of the microgroove barrier; therefore, application of higher capacity microfluidic devices and improvements in MS sensitivity will be crucial for better coverage of the axonal translatome. Diffusion of small molecules (*e.g.* amino acids) between the wells is another critical aspect in the application of dynamic SILAC to microfluidic devices. We experimentally confirmed that diffusion does not take place in three ways: addition of heavy SILAC amino acids into an empty distal well did not lead to any significant detection of SILAC incorporation in the cells cultured in the proximal well, even after prolonged incubation time (Fig. 3, *A*–*C*). In theory, diffusion can also take place intracellularly, after a small molecule enters the cytosol. This was addressed in the experiment where CHX was used to inhibit the protein synthesis (Fig. 4*B*). Addition of CHX to the distal (axonal) well did not have an influence on the protein synthesis in the proximal well, confirming that no measurable intracellular diffusion took place in the time course of analysis. The use of differential dynamic SILAC labeling to study protein transport between cell compartments is complicated by the fact that two processes, protein synthesis and protein transport, are being simultaneously measured. Therefore, the transport of even abundant but low turnover proteins could not be detected in our experimental setup and only limited information on transport rates can be inferred from our data. Finally, we used commercially available dyes that are taken up by cells to stain mitochondria (MitoTracker Deep Red) and neutral lipids (Bodipy) on only one side of the microfluidic devices. We tracked the stained organelles and lipids on either side and could observe only intracellular diffusion/transport in the axons traversing the microgroove (Supplemental Fig. S5, *D*–*F*).

## Data availability

The mass spectrometry proteomics data have been deposited to the ProteomeXchange Consortium *via* the PRIDE (104) partner repository with the dataset identifier: PXD050991 (DIA dataset), PXD051015 (turnover dataset), and PXD051016 (microfluidic devices). The annotated spectra were deposited in MS viewer (105) with the search keys gq4asqc8vd (dataset 2) and gmtoiiyuod (dataset 3.3).

## Supplemental data

This article contains supplemental data.

## Conflicts of interest

The authors declare no competing interests.
