# Supplementary material for: Proteome Dynamics in iPSC-Derived Human Dopaminergic Neurons
Source: Mol Cell Proteomics. 2024 Sep 7;23(10):100838. doi: 10.1016/j.mcpro.2024.100838 (PMC11474371; doi:10.1016/j.mcpro.2024.100838)
Supplement: Supplementary data [file mmc1.docx]

**Proteome dynamics in iPSC-derived human dopaminergic neurons**

Claudia Cavarischia-Rega^1*^, Karan Sharma^2*^, Julia C. Fitzgerald^2#^, Boris Macek^1#^

1) Quantitative Proteomics, Department of Biology, Interfaculty Institute of Cell Biology, University of Tübingen, Germany; 2) Department of Neurodegeneration, Hertie Institute for Clinical Brain Research, University of Tübingen, Germany

**Supplementary Materials**

Supplementary Figure S1: Deep proteome coverage of hDANs using DIA

Supplementary Figure S2: Additional analysis for protein turnover of hDANs

Supplementary Figure S3: Protein turnover of selected complexes and of mitophagy pathway

Supplementary Figure S4: Differential analysis of soma and axons

Supplementary Figure S5: Live fluorescent imaging of hDANs in the microfluidic device.

Supplementary Figure S6: Inhibition of protein synthesis with CHX of selected examples after 72 hours

Supplementary Figure S7: Additional analysis of KIF5 and DYNC1H1

Supplementary Table 1 (S1): Comprehensive coverage of the proteome using DIA

Supplementary Table 2 (S2): Protein turnover dynamics using SILAC

Supplementary Table 3 (S3): Dynamic SILAC to study local synthesis and trafficking of proteins

## SUPPLEMENTARY FIGURE LEGENDS

**Figure S1: Deep proteome coverage of hDANs using DIA**

**A.** Pearson correlation between the three biological replicates based on MS ion intensity. **B.** Total number of identified proteins groups. Mean with error bars (SEM). **C**. Distribution of proteins according to their intensities into five different bins. Mitochondrial proteins are marked in red. **D.** Panther pathways enrichment analysis of identified proteins in the five distinct intensity bins using Fisher exact test (FDR <0.05).

**Figure S2: Additional analysis for protein turnover of hDANs**

**A.** Pearson correlation between the three biological replicates and all six time points based on MS ion intensity. **B.** Distribution of protein half-lives in hDANs ranged from, >1day to >15 days. **C**. Distribution of proteins according to their half-lives into seven different bins. In red are marked mitochondrial proteins. **D.** Panther pathways enrichment analysis of identified proteins in the seven distinct half-life bins using Fisher exact test (FDR <0.05).

**Figure S3: Protein turnover of selected complexes and of mitophagy pathway**

**A.** Schematic illustration of the cytosolic ribosome (left panel), the mitochondrial ribosome (middle panel), and the 26S proteasome (right panel) showing half-lives for individual components of each complex. Proteins are color coded as a gradient from light pink (short half- life) to dark blue-black (long half- life). **B.** Bar graph representing the mean with error bars (SEM) of the half-lives of proteins from large and small subunit of the cytosolic ribosome and of the mitochondrial ribosome (upper panel), and from 19S and 20S subunit of the proteasome (lower panel). Significant *p≤0.05, **p≤ 0.01 and ***p≤ 0.001, t-test. **C.** Schematic illustration of the half-lives of proteins involved in the mitophagy pathway. Proteins are color coded as gradient from light pink (short half- life) to dark blue-black (long half- life).

**Figure S4: Differential analysis of soma and axons**

**A.** Photographs of microfluidic devices of the somatodendritic well and the axonal well from the diffusion experiment on the initial day after seeding, and on the day of harvest. **B.** Photographs of microfluidic devices of the somatodendritic well, the microgroove barrier and the axonal well from the time course experiment on the harvest day. **C**. Principal component analysis based on protein intensity showing the separation of axonal and soma samples, independent of time point and replicates. Clustering of samples are indicated by colored ellipses. **D.** Normalized intensity of MAP1B, a known axonal marker, from all times points and replicates from somatodendritic well (green) and the axonal well (red) are shown. ***p≤ 0.001, t-test. **E.** Scatter plot of log_2_-transformed ratios for proteins quantified in the axonal samples versus in the soma samples. Proteins marked in orange are those annotated as axonal according to GO terms, and those that have a 4x fold higher abundance in the axon are numbered and listed. **F.** Venn diagram of protein hits with a 4x fold higher abundance (enriched) in axon compared to soma, proteins exclusively quantified in the axon and all the genes annotated as axonal proteins according to GO terms. **G.** Panther GOCC enrichment analysis of identified proteins as axonal enriched and exclusively quantified proteins using Fisher exact test (FDR <0.05).

**Figure S5: Live fluorescent imaging of hDANs in the microfluidic device**

**A.** Representative images of the somatodendritic well stained with TH. Scale bar: 500 μm. **B.** Representative images of the axonal well stained with TH. Scale bar: 500 μm. **C.** Intensity of TH, a known dopaminergic neurons marker, TPH2, a known serotonergic neurons marker, GAD1, a known GABAergic neurons marker, and CHAT, a known cholinergic neurons marker. ***p≤ 0.001, t-test **D.** Schematic showing the experimental setup. Bodipy stains neutral lipids and was applied on the soma side. MitoTracker stains mitochondria and was applied on the axonal side. The dyes were added in fresh media and not removed. **E.** Live cell imaging (x4 magnification) at 1.5 and 24h following addition of the dyes show only intracellular staining on the respective sides of the device and some mitochondrial migration in the axons spanning the microgroove. The scale bar is 100µm. **F.** Live cell imaging (x20 magnification) of the soma side and microgroove show the stained mitochondria are transported via the axons towards the soma of cells near to the microgroove barrier after 24h. The scale bar is 100µm. **G.** Representative images of hDANs stained with GAP43 and Hoechst. Scale bar: 50 μm. **H.** Representative images of hDANs stained with STMN2 and Hoechst. Scale bar: 50 μm **I.** Representative images of hDANs stained with DHX30 and Hoechst. Scale bar: 50 μm

**Figure S6: Inhibition of protein synthesis with CHX of selected examples after 72 hours**

**A-F.** Label incorporation of the medium-heavy and heavy label at 72 hours after addition of CHX in the somatodendritic well and the axonal well of KIF5C, KLC1, DHX30, ADAR, SEC24A and RAB11B in soma and in axon. The label incorporation from the turnover dataset is also depicted. All the bar graphs represent mean with error bars (SEM) from 3 biological replicates.

**Figure S7: Additional analysis of KIF5 and DYNC1H1**

**A.** Label incorporation curves of the three different isoforms of KIF5 in the turnover dataset, with their half-lives. **B.** Intensity of the three different isoforms of KIF5 in the microfluidic devices at each time point from the time course experiment. **C.** Label incorporation curves of the heavy and medium-heavy label of DYNC1H1 in soma and the label incorporation curve of DYNC1H1 in the turnover dataset. **D.** Label incorporation of the medium-heavy and heavy label at 72 hours after addition of CHX in the somatodendritic well and the axonal well of DYNC1H1 in soma and in axon. The label incorporation from the turnover dataset is also depicted. All the bar graphs represent mean with error bars (SEM) from 3 biological replicates.
