# Supplementary figures and images for: Proteome Dynamics in iPSC-Derived Human Dopaminergic Neurons
Source: Mol Cell Proteomics. 2024 Sep 7;23(10):100838. doi: 10.1016/j.mcpro.2024.100838 (PMC11474371; doi:10.1016/j.mcpro.2024.100838)

A)

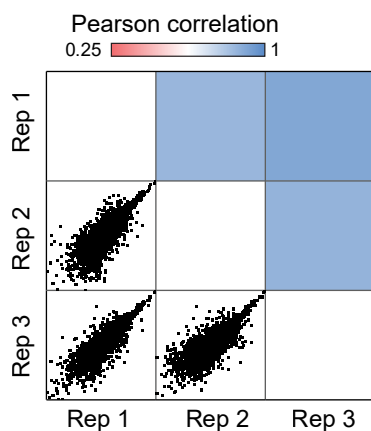

B)

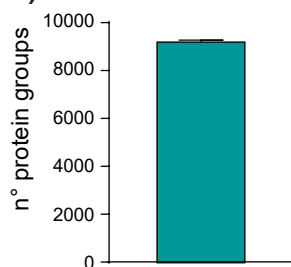

C)

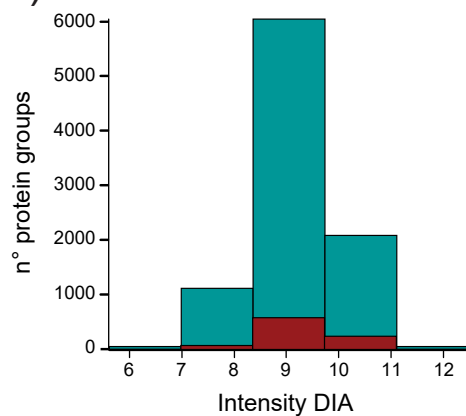

D)

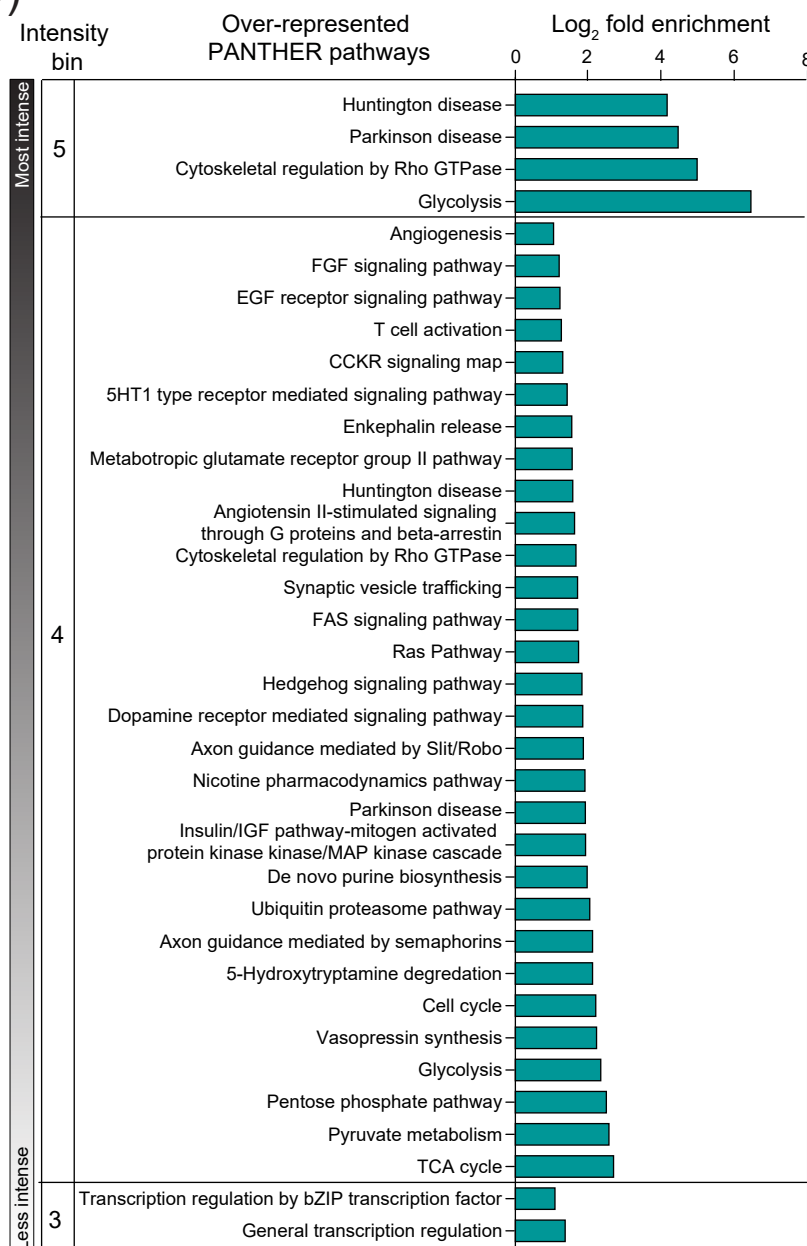

Supplement: Fig S1 [file mmc2.pdf]

A)

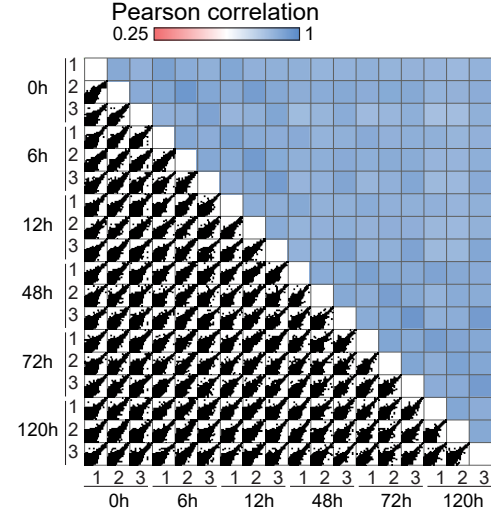

B)

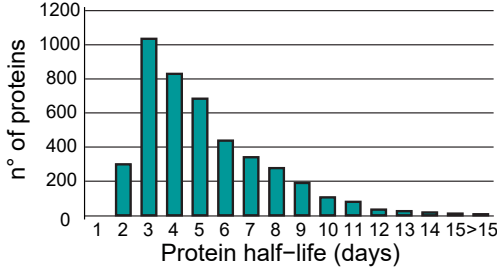

C)

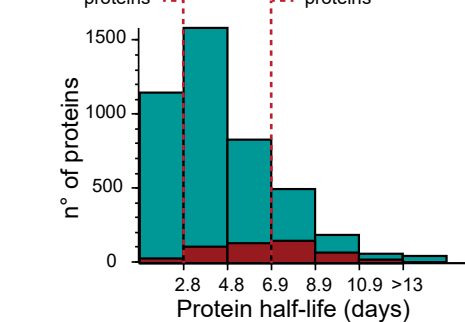

D)

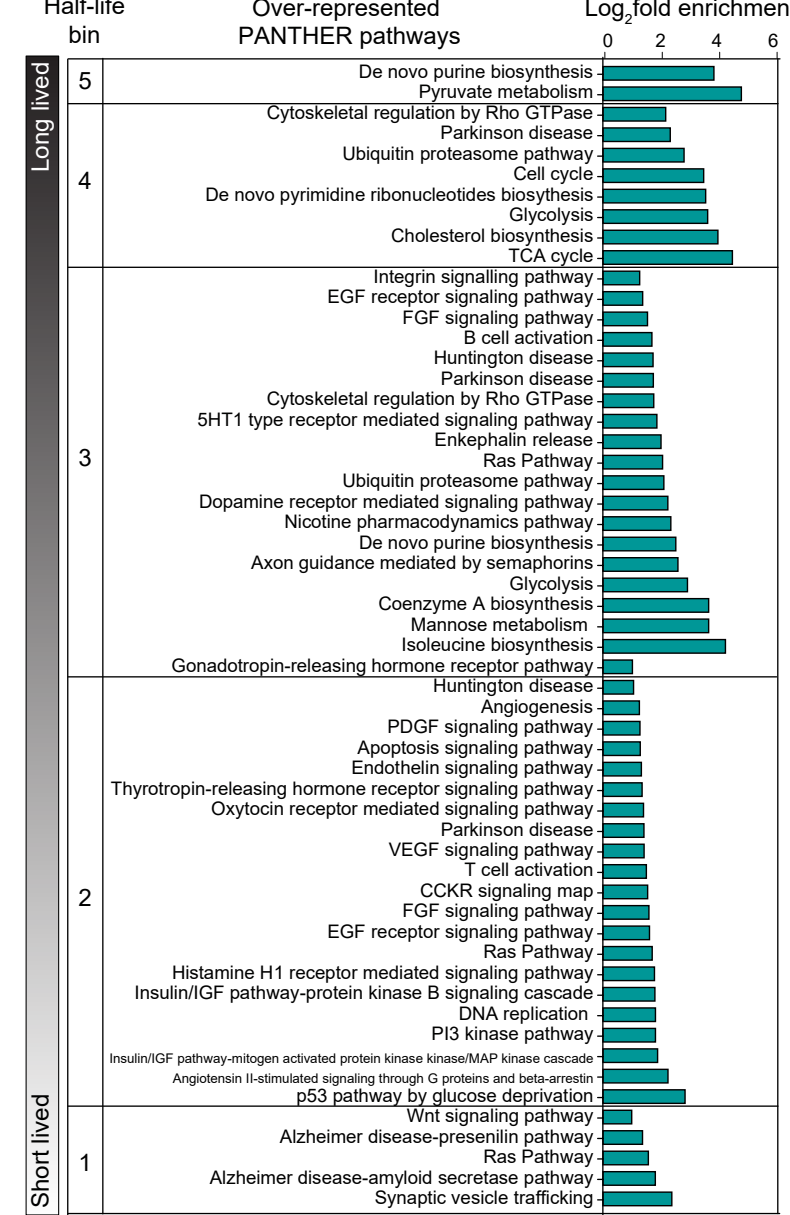

Supplement: Fig S2 [file mmc3.pdf]

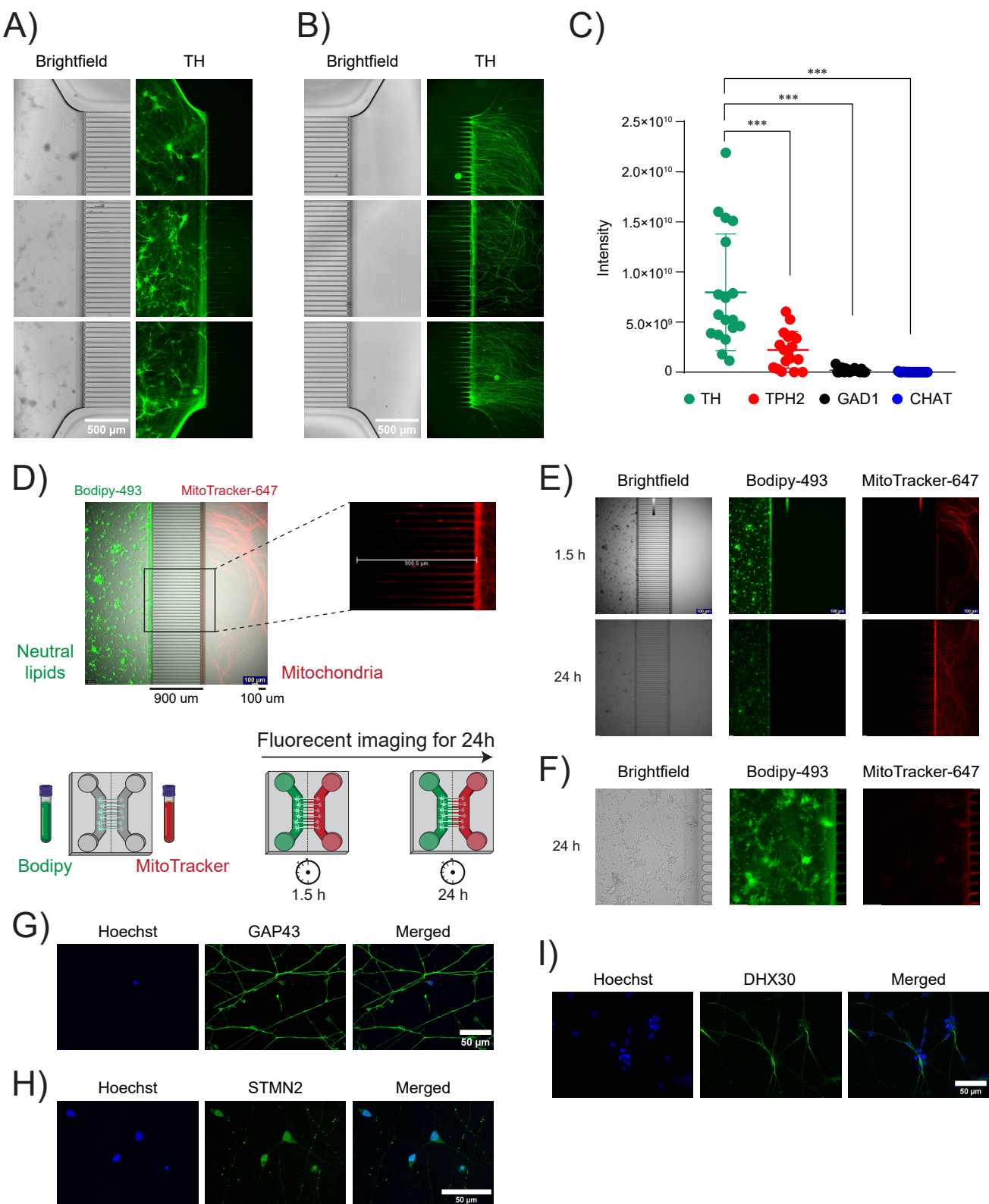

Supplement: Fig S5 [file mmc6.pdf]

A)

KIF5C at 72h

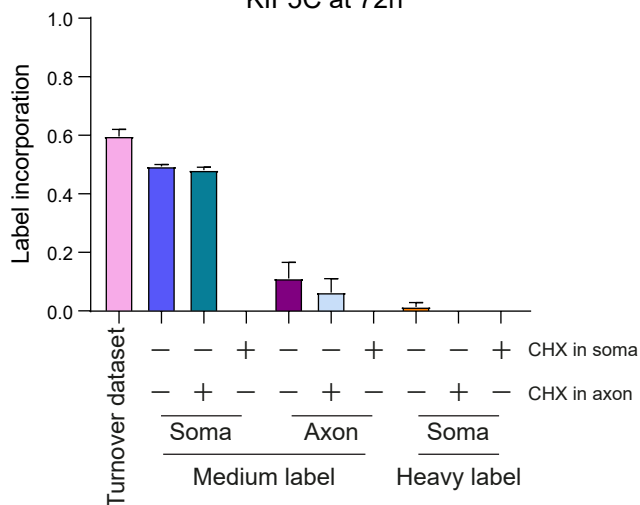

B)

KLC1 at 72h

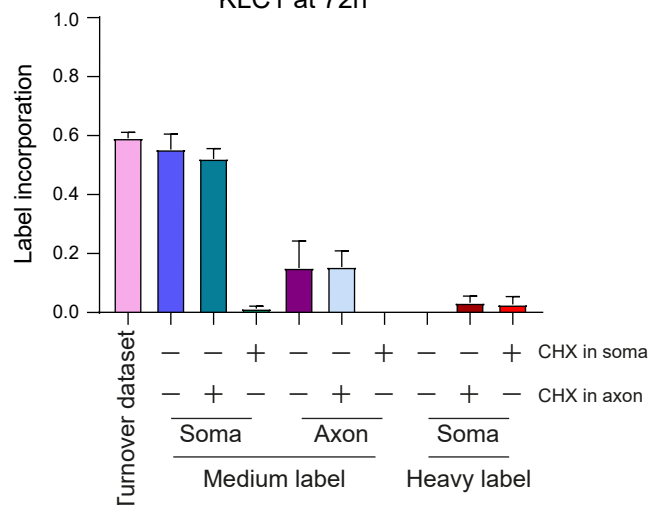

C)

DHX30 at 72h

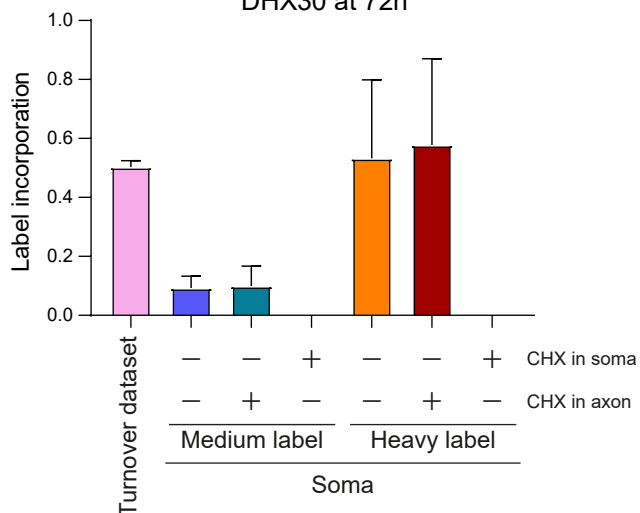

D)

ADAR at 72h

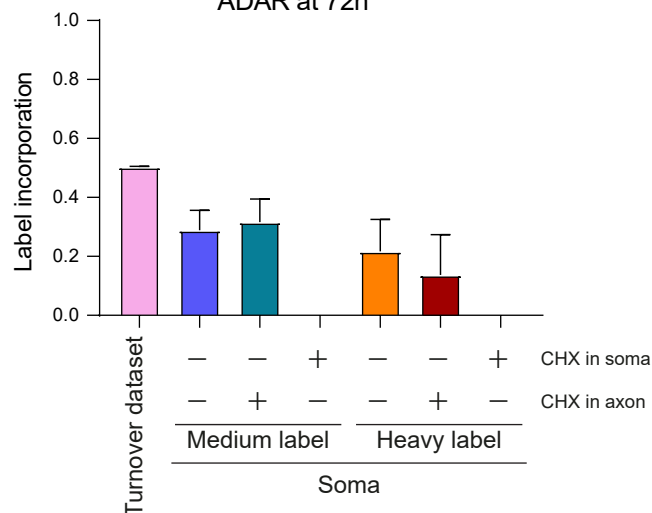

E)

SEC24A at 72h

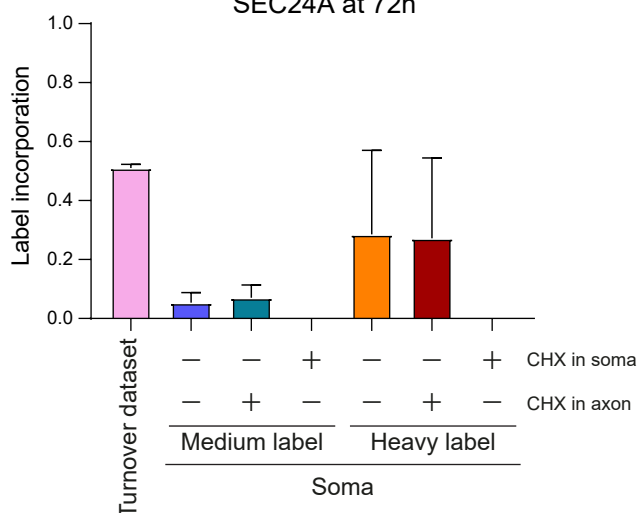

F)

RAB11B at 72h

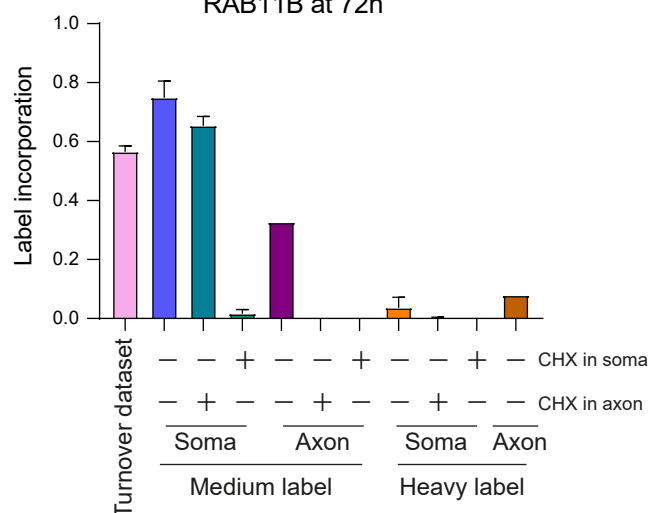

Supplement: Fig S6 [file mmc7.pdf]

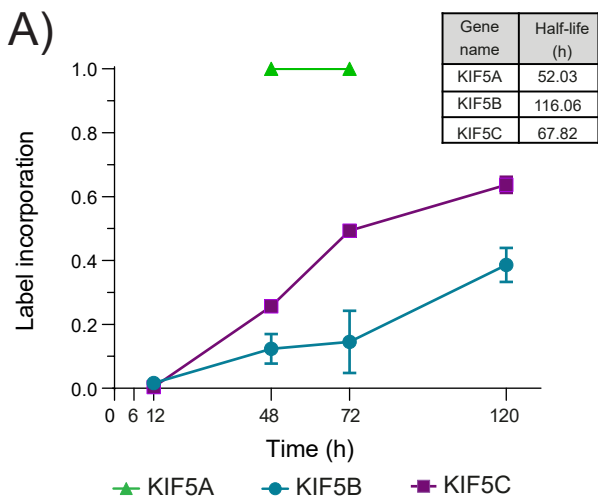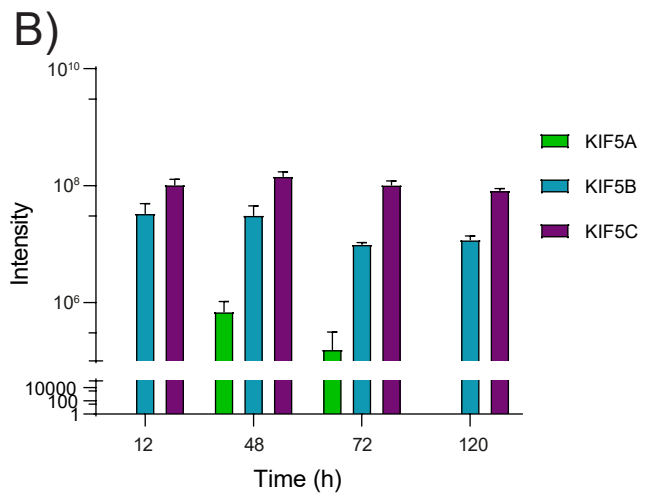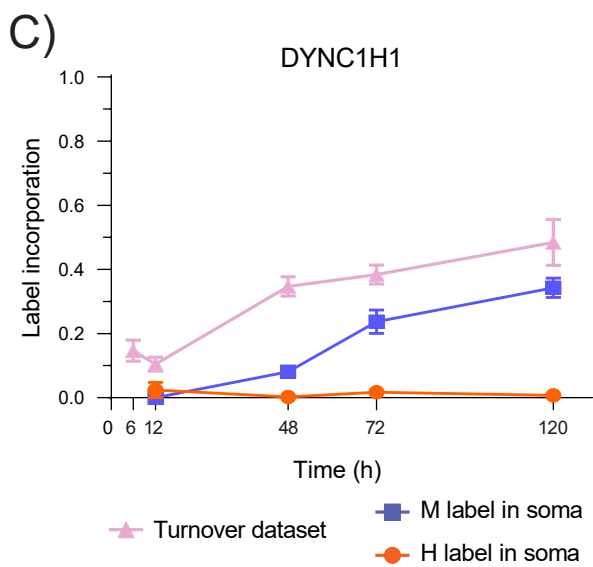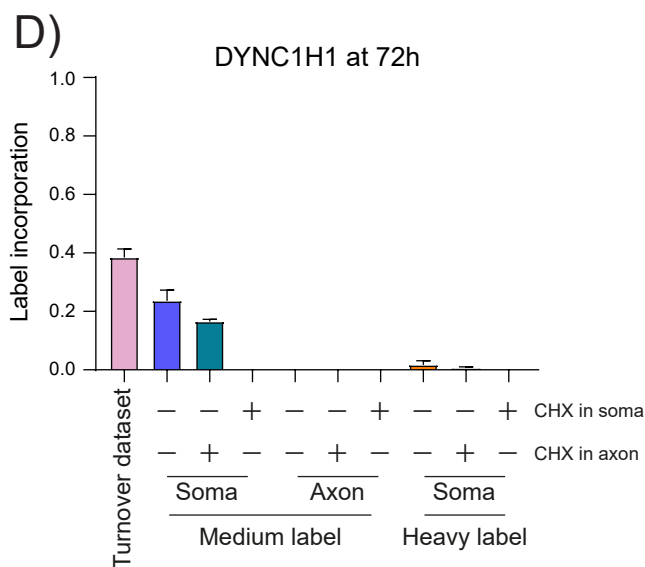

Supplement: Fig S7 [file mmc8.pdf]
