# Supplementary material for: Proteome Dynamics in iPSC-Derived Human Dopaminergic Neurons
Source: Mol Cell Proteomics. 2024 Sep 7;23(10):100838. doi: 10.1016/j.mcpro.2024.100838 (PMC11474371; doi:10.1016/j.mcpro.2024.100838)
Supplement: Fig S3 [file mmc4.pdf]

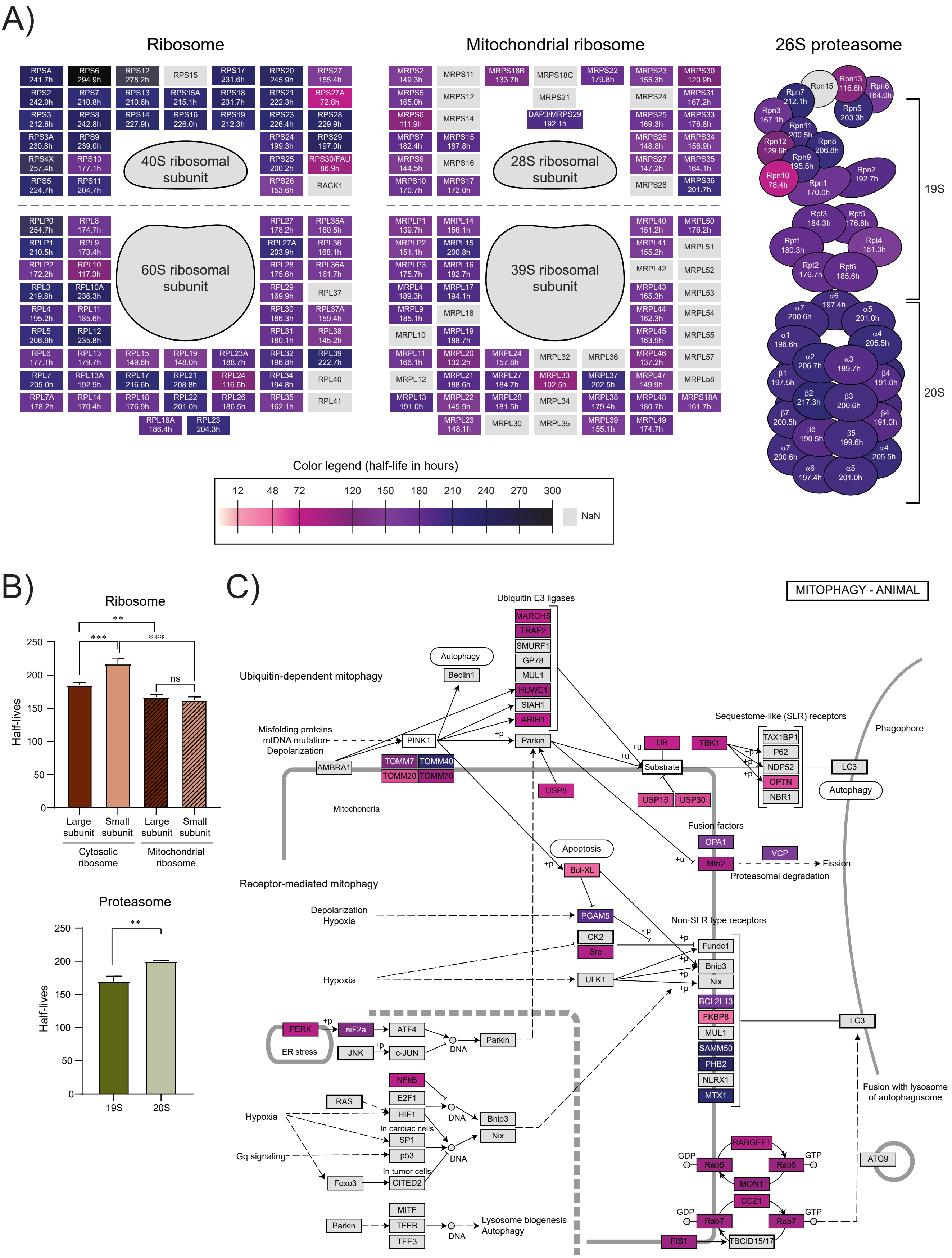
A)
Ribosome
RPSA 241.7h
RPS6 294.9h
RPS12 278.2h
RPS15
RPS17 231.6h
RPS20 245.9h
RPS27 155.4h
RPS2 242.0h
RPS7 210.8h
RPS13 210.6h
RPS15A 215.1h
RPS18 231.7h
RPS21 222.3h
RPS27A 72.8h
RPS3 212.6h
RPS8 242.8h
RPS14 227.9h
RPS16 226.0h
RPS19 212.3h
RPS23 226.4h
RPS28 229.9h
RPS3A 230.8h
RPS9 239.0h
RPS24 199.3h
RPS29 197.0h
RPS4X 257.4h
RPS10 177.1h
40S ribosomal subunit
RPS25 200.2h
RPS30/FAU 86.9h
RPS5 224.7h
RPS11 204.7h
RPS26 153.6h
RACK1
RPLP0 254.7h
RPL8 174.7h
60S ribosomal subunit
RPL27 178.2h
RPL35A 160.5h
RPLP1 210.5h
RPL9 173.4h
RPL27A 203.9h
RPL36 168.1h
RPLP2 172.2h
RPL10 117.3h
RPL28 175.6h
RPL36A 161.7h
RPL3 219.8h
RPL10A 236.3h
RPL29 169.9h
RPL37
RPL4 195.2h
RPL11 185.6h
RPL30 186.3h
RPL37A 159.4h
RPL5 206.9h
RPL12 235.8h
RPL31 180.1h
RPL38 145.2h
RPL6 177.1h
RPL13 179.7h
RPL15 149.6h
RPL19 148.0h
RPL23A 188.7h
RPL32 196.8h
RPL39 222.7h
RPL7 205.0h
RPL13A 192.9h
RPL17 216.6h
RPL21 208.8h
RPL24 116.6h
RPL34 194.8h
RPL40
RPL7A 178.2h
RPL14 170.4h
RPL18 176.9h
RPL22 201.0h
RPL26 186.5h
RPL35 162.1h
RPL41
RPL18A 186.4h
RPL23 204.3h
Mitochondrial ribosome
MRPS2 149.3h
MRPS11
MRPS18B 133.7h
MRPS18C
MRPS22 179.8h
MRPS23 155.3h
MRPS30 120.9h
MRPS5 165.0h
MRPS12
MRPS21
MRPS24
MRPS31 167.2h
MRPS6 111.9h
MRPS14
DAP3/MRPS29 192.1h
MRPS25 169.3h
MRPS33 176.8h
MRPS7 182.4h
MRPS15 187.8h
MRPS26 148.8h
MRPS34 156.9h
MRPS9 144.5h
MRPS16
28S ribosomal subunit
MRPS27 147.2h
MRPS35 164.1h
MRPS10 170.7h
MRPS17 172.0h
MRPS28
MRPS36 201.7h
MRPLP1 139.7h
MRPL14 156.1h
39S ribosomal subunit
MRPL40 151.2h
MRPL50 176.2h
MRPLP2 151.1h
MRPL15 200.8h
MRPL41 155.2h
MRPL51
MRPLP3 175.7h
MRPL16 182.7h
MRPL42
MRPL52
MRPL4 189.3h
MRPL17 194.1h
MRPL43 165.3h
MRPL53
MRPL9 185.1h
MRPL18
MRPL44 162.3h
MRPL54
MRPL10
MRPL19 188.7h
MRPL45 163.9h
MRPL55
MRPL11 166.1h
MRPL20 132.2h
MRPL24 157.8h
MRPL32
MRPL36
MRPL46 137.2h
MRPL57
MRPL12
MRPL21 188.6h
MRPL27 184.7h
MRPL33 102.5h
MRPL37 202.5h
MRPL47 149.9h
MRPL58
MRPL13 191.0h
MRPL22 145.9h
MRPL28 181.5h
MRPL34
MRPL38 179.4h
MRPL48 180.7h
MRPS18A 161.7h
MRPL23 148.1h
MRPL30
MRPL35
MRPL39 155.1h
MRPL49 174.7h
26S proteasome
Rpn15
Rpn13 116.6h
Rpn6 164.0h
Rpn7 212.1h
Rpn3 167.1h
Rpn5 203.3h
Rpn11 200.5h
Rpn12 129.6h
Rpn8 206.8h
Rpn9 195.5h
Rpn10 78.4h
Rpn2 192.7h
Rpn1 170.0h
Rpt3 184.3h
Rpt5 176.8h
Rpt1 180.3h
Rpt4 161.3h
Rpt2 176.7h
Rpt6 185.6h
19S
α6 197.4h
α7 200.6h
α5 201.0h
α1 196.6h
α4 205.5h
α2 206.7h
α3 189.7h
β1 197.5h
β4 191.0h
β2 217.3h
β3 200.6h
β7 200.5h
β4 191.0h
β6 190.5h
β5 199.6h
α7 200.6h
α4 205.5h
α6 197.4h
α5 201.0h
20S
Color legend (half-life in hours)
12 48 72 120 150 180 210 240 270 300
NaN
B)
Ribosome
**
***
***
ns
Half-lives
250
200
150
100
50
0
Large subunit
Small subunit
Large subunit
Small subunit
Cytosolic ribosome
Mitochondrial ribosome
Proteasome
**
Half-lives
250
200
150
100
50
0
19S
20S
C)
MITOPHAGY - ANIMAL
Ubiquitin E3 ligases
MARCH5
TRAF2
SMURF1
GP78
MUL1
HUWE1
SIAH1
ARIH1
Autophagy
Beclin1
Ubiquitin-dependent mitophagy
Misfolding proteins
mtDNA mutation
Depolarization
PINK1
+p
Parkin
AMBRA1
TOMM7
TOMM40
TOMM20
TOMM70
+u
UB
+u
Substrate
TBK1
+p
+p
+p
Sequestome-like (SLR) receptors
TAX1BP1
P62
NDP52
OPTN
NBR1
LC3
Autophagy
Phagophore
USP8
USP15
USP30
Mitochondria
Fusion factors
OPA1
VCP
Apoptosis
+u
Mfn2
Fission
+p
Bcl-XL
Proteasomal degradation
Receptor-mediated mitophagy
Depolarization
Hypoxia
PGAM5
- p
Non-SLR type receptors
CK2
Src
+p
Fundc1
+p
Bnip3
+p
Hypoxia
ULK1
Nix
+p
BCL2L13
FKBP8
MUL1
SAMM50
PHB2
NLRX1
MTX1
LC3
PERK
+p
eiF2a
ATF4
ER stress
JNK
+p
c-JUN
DNA
Parkin
NFkB
RAS
E2F1
HIF1
DNA
Bnip3
Hypoxia
In cardiac cells
SP1
Nix
Gq signaling
p53
DNA
In tumor cells
Foxo3
CITED2
Fusion with lysosome of autophagosome
RABGEF1
GDP
Rab5
Rab5
GTP
MON1
ATG9
CCZ1
GDP
Rab7
Rab7
GTP
MITF
Parkin
TFEB
DNA
Lysosome biogenesis
Autophagy
TFE3
FIS1
TBCID15/17
