# Supplementary material for: Proteome Dynamics in iPSC-Derived Human Dopaminergic Neurons
Source: Mol Cell Proteomics. 2024 Sep 7;23(10):100838. doi: 10.1016/j.mcpro.2024.100838 (PMC11474371; doi:10.1016/j.mcpro.2024.100838)
Supplement: Fig S4 [file mmc5.pdf]

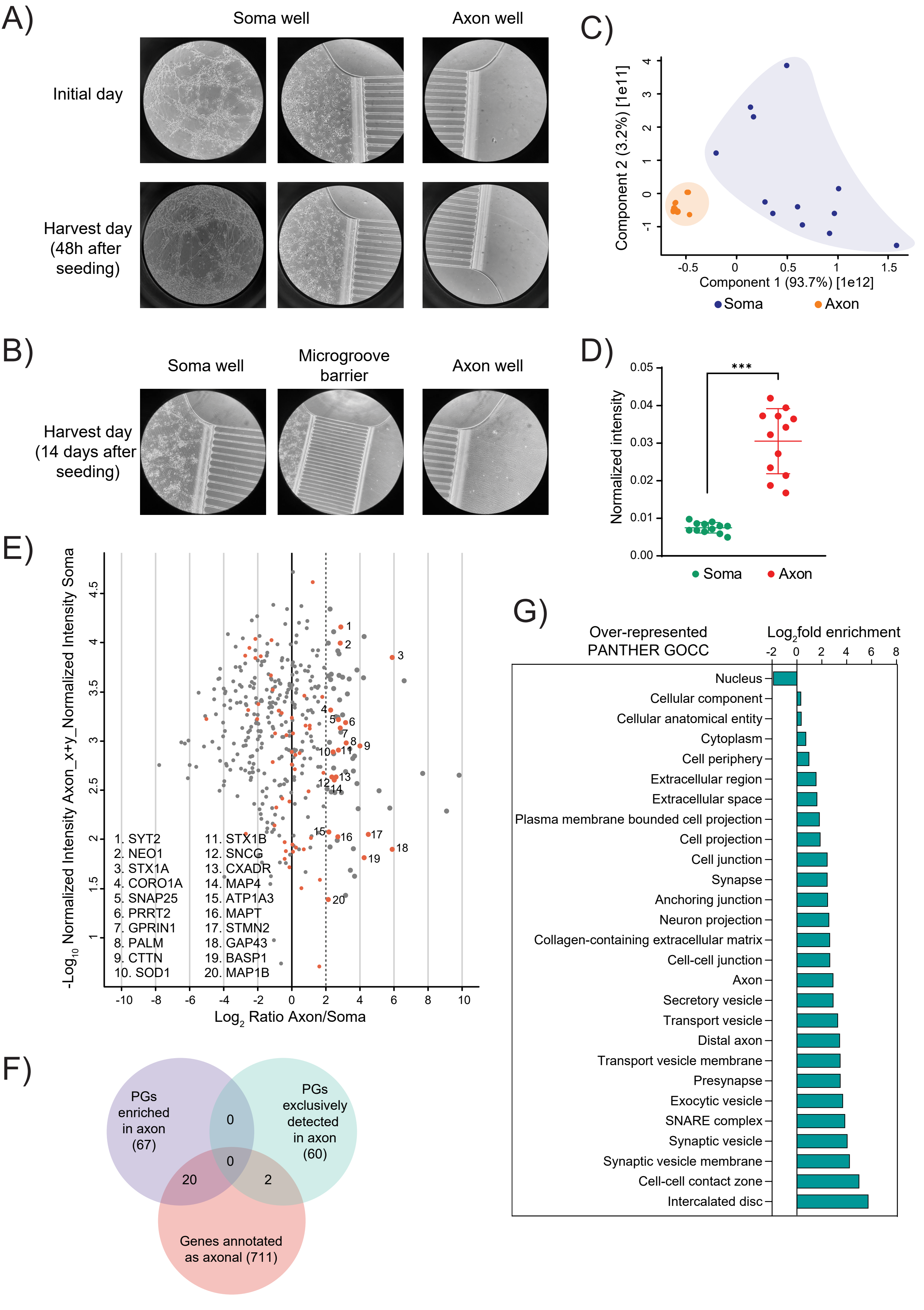
A)
Soma well
Axon well
Initial day
Harvest day
(48h after
seeding)
B)
Soma well
Microgroove
barrier
Axon well
Harvest day
(14 days after
seeding)
C)
Component 2 (3.2%) [1e11]
Component 1 (93.7%) [1e12]
Soma
Axon
D)
Normalized intensity
***
Soma
Axon
E)
-Log10 Normalized Intensity Axon_x+y_Normalized Intensity Soma
Log2 Ratio Axon/Soma
1. SYT2
2. NEO1
3. STX1A
4. CORO1A
5. SNAP25
6. PRRT2
7. GPRIN1
8. PALM
9. CTTN
10. SOD1
11. STX1B
12. SNCG
13. CXADR
14. MAP4
15. ATP1A3
16. MAPT
17. STMN2
18. GAP43
19. BASP1
20. MAP1B
F)
PGs
enriched
in axon
(67)
PGs
exclusively
detected
in axon
(60)
Genes annotated
as axonal (711)
0
0
20
2
G)
Over-represented
PANTHER GOCC
Log2fold enrichment
Nucleus
Cellular component
Cellular anatomical entity
Cytoplasm
Cell periphery
Extracellular region
Extracellular space
Plasma membrane bounded cell projection
Cell projection
Cell junction
Synapse
Anchoring junction
Neuron projection
Collagen-containing extracellular matrix
Cell-cell junction
Axon
Secretory vesicle
Transport vesicle
Distal axon
Transport vesicle membrane
Presynapse
Exocytic vesicle
SNARE complex
Synaptic vesicle
Synaptic vesicle membrane
Cell-cell contact zone
Intercalated disc
